# Impact of Topical Interventions on the Vaginal Microbiota and Metabolome in Postmenopausal Women: A Secondary Analysis of a Randomized Clinical Trial

**DOI:** 10.1001/jamanetworkopen.2022.5032

**Published:** 2022-03-30

**Authors:** Sujatha Srinivasan, Xing Hua, Michael C. Wu, Sean Proll, D. J. Valint, Susan D. Reed, Katherine A. Guthrie, Andrea Z. LaCroix, Joseph C. Larson, Robert Pepin, Shalender Bhasin, Daniel Raftery, David N. Fredricks, Caroline M. Mitchell

**Affiliations:** 1 Vaccine and Infectious Disease Division, Fred Hutchinson Cancer Research Center, Seattle, Washington; 2 Public Health Sciences Division, Fred Hutchinson Cancer Research Center, Seattle, Washington; 3 Department of Obstetrics and Gynecology, University of Washington, Seattle; 4 Herbert Wertheim School of Public Health, University of California, San Diego; 5 Department of Anesthesia & Pain Medicine, University of Washington, Seattle; 6 Research Program in Men’s Health, Aging and Metabolism, Department of Medicine, Boston Claude D. Pepper Older Americans Independence Center, Brigham and Women’s Hospital, Boston, Massachusetts; 7 Department of Medicine, University of Washington, Seattle; 8 Vincent Center for Reproductive Biology, Massachusetts General Hospital, Boston

## Abstract

**Importance:**

Postmenopausal women with genitourinary symptoms of menopause are often prescribed vaginal estradiol or moisturizer for symptom improvement, but the impact of these treatments on the local microenvironment is poorly understood.

**Objective:**

To compare changes in the vaginal microbiota, metabolome, and pH among women using low-dose vaginal estradiol tablet or low pH moisturizer gel for 12-weeks vs low pH placebo.

**Design, Setting, and Participants:**

This is a post hoc prespecified secondary analysis of a 12-week multicenter randomized clinical trial among postmenopausal women with moderate to severe genitourinary symptoms. Women were enrolled between April 2016 and February 2017; final follow-up visits occurred in April 2017. Data were analyzed from November 2018 to July 2021.

**Interventions:**

Ten-μg vaginal estradiol plus placebo gel vs placebo tablet plus vaginal moisturizer vs dual placebo.

**Main Outcomes and Measures:**

The main outcome measures were changes in the diversity and composition of the vaginal microbiota, changes in the metabolome, and pH.

**Results:**

Of 302 postmenopausal women from the parent trial, 144 women (mean [SD] age, 61 [4] years) were included in this analysis. After 12 weeks, the microbiota was dominated with *Lactobacillus* and *Bifidobacterium* communities among 36 women (80%) in the estradiol group, compared with 16 women (36%) using moisturizer and 13 women (26%) using placebo (*P* < .001). The composition of vaginal fluid metabolites also varied after 12-weeks among women in the estradiol group with significant changes in 90 of 171 metabolites measured (53%) (*P* < .001), including an increase in lactate. The 12-week pH among women in the estradiol group was lower vs placebo (median [IQR] pH, 5 [4.5-6.0] vs 6 [5.5-7.0]; *P* = .005) but not the moisturizer group vs placebo (median [IQR] pH, 6 [5.5-6.5]; *P* = .28). There was a decrease in pH from baseline to 12-weeks within the moisturizer (median [IQR] pH, 7 [6.0-7.5] vs 6 [5.5-6.5]; *P* < .001) and placebo (median [IQR] pH, 7 [7.0-7.5] vs 6 [5.5-7.0]; *P* < .001) groups. Women with high-diversity bacterial communities at baseline exhibited greater median change in pH compared with women with low-diversity communities (median [IQR] change, −1 [−2 to −0.5] vs −0.3 [−1.1 to 0], *P* = .007).

**Conclusions and Relevance:**

This secondary analysis of a randomized clinical trial found that use of vaginal estradiol tablets resulted in substantial changes in the vaginal microbiota and metabolome with a lowering in pH, particularly in women with high-diversity bacterial communities at baseline. Low pH moisturizer or placebo did not significantly impact the vaginal microbiota or metabolome despite lowering the vaginal pH. Estradiol use may offer additional genitourinary health benefits to postmenopausal women.

**Trial Registration:**

ClinicalTrials.gov Identifier: NCT02516202

## Key Points

**Question:**

What are the effects of estradiol or vaginal moisturizer on the vaginal microbiota, metabolome, and pH after 12-week treatment in postmenopausal women?

**Findings:**

In this secondary analysis of 144 participants in a randomized clinical trial of postmenopausal women with moderate to severe vulvovaginal symptoms, women using vaginal 10 μg estradiol tablet demonstrated larger changes in the vaginal microbiota and metabolome compared with women using vaginal moisturizer or placebo, despite a decrease in pH within each intervention group.

**Meaning:**

These findings suggest that treatment of genitourinary symptoms of menopause with topical estradiol changes the vaginal microenvironment in ways that may promote genitourinary health.

## Teaser

This secondary analysis of a random clinical trial compares changes in the vaginal microbiota, metabolome, and pH among women using a vaginal estradiol tablet, moisturizer, or placebo.

## Introduction

In the Menopause Strategies–Finding Lasting Answers for Symptoms and Health (MsFLASH) trial network’s Vaginal Health Trial of treatment for moderate to severe vaginal symptoms of menopause, no significant difference in symptom reduction was seen between estradiol vaginal tablet or vaginal moisturizer and placebo: all 3 groups had similar improvement.^1^ While both hormonal and nonhormonal treatments have been shown to improve postmenopausal vaginal symptoms,^1,2,3,4,5,6^ the lack of difference between estradiol and placebo was surprising. The placebo gel used in the trial had high lubricity and low pH, while the moisturizer also had low pH; both properties may contribute to decreased symptoms via biological effects that differ from estradiol. Some interpreted these findings as a statement that estrogen treatment was ineffective, rather than a recognition that different types of interventions may reduce postmenopausal vaginal discomfort.

The Food and Drug Administration (FDA) guidance for outcome measures in clinical trials of treatment for moderate to severe genitourinary syndrome of menopause (GSM) recommends 3 coprimary end points: change in most bothersome symptom (MBS) severity, pH, and vaginal maturation index (VMI).^7^ In our primary analysis, more participants in the estrogen group had pH less than 5 and more than 5% superficial cells on VMI than placebo at 12 weeks, although the placebo gel was acidic.^1^ The regulatory guidance weights these changes in the vaginal mucosal environment equally with improvement in MBS, but it is unclear whether these mucosal changes reflect any additional benefits to genitourinary health beyond symptom improvement.

We conducted a subset post hoc analysis of participants from the MsFLASH Vaginal Health Trial to evaluate the effect of topical interventions for GSM on the microbiota and metabolome, and to assess whether larger changes in pH and VMI in the estradiol group reflect other changes in the vaginal microbiota and metabolome that might impact genitourinary health.

## Methods

This secondary analysis of a randomized clinical trial was approved by the institutional review board at Fred Hutchinson Cancer Research Center. All participants provided written informed consent. The primary trial and this prespecified post hoc secondary analysis followed the Consolidated Standards of Reporting Trials (CONSORT) reporting guideline. The primary trial protocol and statistical analysis plan are provided in Supplement 1.

### Study Design and Population

The MsFLASH Vaginal Health Trial was a multicenter, double-blind randomized clinical trial of vaginal estradiol tablet 0.01 mg (plus placebo gel) or vaginal moisturizing gel (plus placebo tablet) vs dual placebo in 302 postmenopausal women with moderate to severe vulvovaginal discomfort.^1^ Women were enrolled between April 2016 and February 2017; final follow-up visits occurred in April 2017. The primary outcome was change over 12-weeks in severity of MBS selected by participants at enrollment, including pain with penetration, vaginal dryness, and vulvovaginal irritation, itching, or pain. Symptom severity was rated from 0 to 3, signifying none, mild, moderate, or severe.

A subset of 144 women eligible for this post hoc analysis used at least 80% of study product doses and were not diagnosed with yeast, bacterial vaginosis (BV), or trichomoniasis by culture or wet mount. We randomly selected 15 eligible participants from each group to evaluate changes in the vaginal microbiota and metabolome in response to treatment. We also selected 20 eligible participants from each treatment group who experienced a decrease in MBS of at least 2 points from enrollment to 12 weeks and matched them with 20 eligible participants with a decrease in MBS of 1 point or less by treatment group, race (dichotomized as White or other race, including Black, American Indian, Asian, or Pacific Islander), and MBS selection (pain with vaginal penetration or other), and were frequency-matched by study site, age, and baseline MBS severity. The 2 selection processes were not mutually exclusive. Race was self-identified by the trial participants. Race was included as a factor in matching participants in higher and lower response categories to reduce heterogeneity across the response groups.

At enrollment, 4 weeks, and 12 weeks, vaginal swabs were collected for pH, VMI, microbiota, and metabolomic analyses. Primary outcome measures included changes in diversity and composition of the vaginal microbiota, composition of small molecule metabolites, and pH within the estradiol and moisturizer groups vs placebo across 12-weeks. Microbiota and metabolome characterization details are provided in the eMethods in Supplement 2. Vaginal pH was measured using pH paper. We evaluated change in VMI and symptom improvement when we stratified women based on the diversity of their bacterial communities at baseline. Smears stained with the standard Papanicolau stain were used to evaluate VMI and defined as less than 5% or 5% or greater superficial cells. Symptom improvement was measured as change in the symptom severity scale, and clinically relevant improvement was defined as a decrease of at least 2 points in MBS severity.

### Molecular Methods

The vaginal microbiota was characterized by 16S rRNA gene sequencing; taxonomy was assigned to sequence variants by placing on a custom phylogenetic tree.^8,9,10^ Sequences have been deposited in the National Center for Biotechnology Information Sequence Read Archive (PRJNA788936). Further details are provided in the eMethods in Supplement 2.

### Metabolomic Profiling

Broad-based metabolomic profiling was performed on a liquid-chromatography mass spectrometry (LC-MS/MS) platform at Northwest Metabolomics Research Center.^11^ Further details are provided in the eMethods in Supplement 2.

### Estradiol Concentrations in Serum Samples

Serum estradiol levels were measured at the Brigham Research Assay Core Laboratory using LC-MS/MS on the Triple Quad 5500+ system (SCIEX).^12,13^ Further details are provided in the eMethods in Supplement 2.

### Statistical Analysis

Sequence data were normalized using centered-log-ratio transformation.^14^ We used the Shannon Diversity Index (SDI) to calculate α diversity, and Aitchison distances were used to estimate β diversity. The metabolome data were quantile normalized,^15^ with missing values imputed to the minimum detected value per metabolite divided by √2 and log2-transformed. Changes in the microbiota and metabolome were evaluated across 12 weeks using linear mixed models with the placebo group as the referent and incorporating visit- and participant-specific random effects. Linear mixed models were adjusted for age and time since menopause. False discovery rate correction was conducted using the Benjamini-Hochberg method. Two-sided *P* = .05 and *q* < .20 were considered nominally statistically significant. Correlations among and between metabolite and microbiota abundances were assessed using Spearman rank correlation. Euclidean distances were used for unsupervised hierarchical clustering of microbiota and metabolite data. All statistical analyses were conducted using R version 3.6.0 (R Project for Statistical Computing). Extension packages used included gplots, vegan, MiRKAT, lme4 and ade4. Data were analyzed from November 2018 to July 2021.

## Results

### Study Population

Of 302 postmenopausal women from the parent trial, 144 women (mean [SD] age, 61 [4] years) were included in this analysis. Demographic characteristics of participants in this post hoc secondary analysis were similar to those of the broader original trial population. Most participants were White (129 participants [90%]), 6 participants (4%) were Black, and 9 participants (6%) identified as another race (including American Indian, Asian or Pacific Islander, or unknown) (Table). By design, distribution of women by study group was roughly equivalent: 45 women (31%) were in the estradiol with placebo gel group, 48 women (33%) were in the moisturizer and placebo tablet group, and 51 women (35%) were in the dual placebo group.

**Table.** Baseline Demographic Characteristics of Participants Included in Subset Analysis

Characteristic	Participants, No. (%)		
	Estradiol + placebo gel (n = 45)	Moisturizer + placebo tablet (n = 48)	Dual placebo (n = 51)
Age at screening, mean (SD), y	61 (4)	60 (4)	61 (5)
Race			
Black	5 (11)	1 (2)	0 (0)
White	37 (82)	43 (90)	49 (96)
Other^a^	3 (7)	4 (8)	2 (4)
MBS			
Pain with sex	19 (42)	32 (67)	28 (55)
Dryness	11 (24)	8 (17)	14 (27)
Itch, burn, or irritation	15 (33)	8 (17)	9 (18)
Vaginal maturation index, <5% superficial cells	37 (82)	37 (77)	40 (78)
pH, mean (SD)	6.8 (1.0)	6.6 (1.1)	6.9 (0.9)
Subsequent MBS symptom improvement ≥2 points	24 (53)	21 (44)	25 (49)

Abbreviation: MBS, most bothersome symptom.

^a^ Other race includes 2 American Indian individuals, 6 Asian or Pacific Islander individuals, and 1 unknown individual with unknown race.

### Impact of Moisturizer and Estradiol Treatment on the Vaginal Microbiota

**Figure 1.**
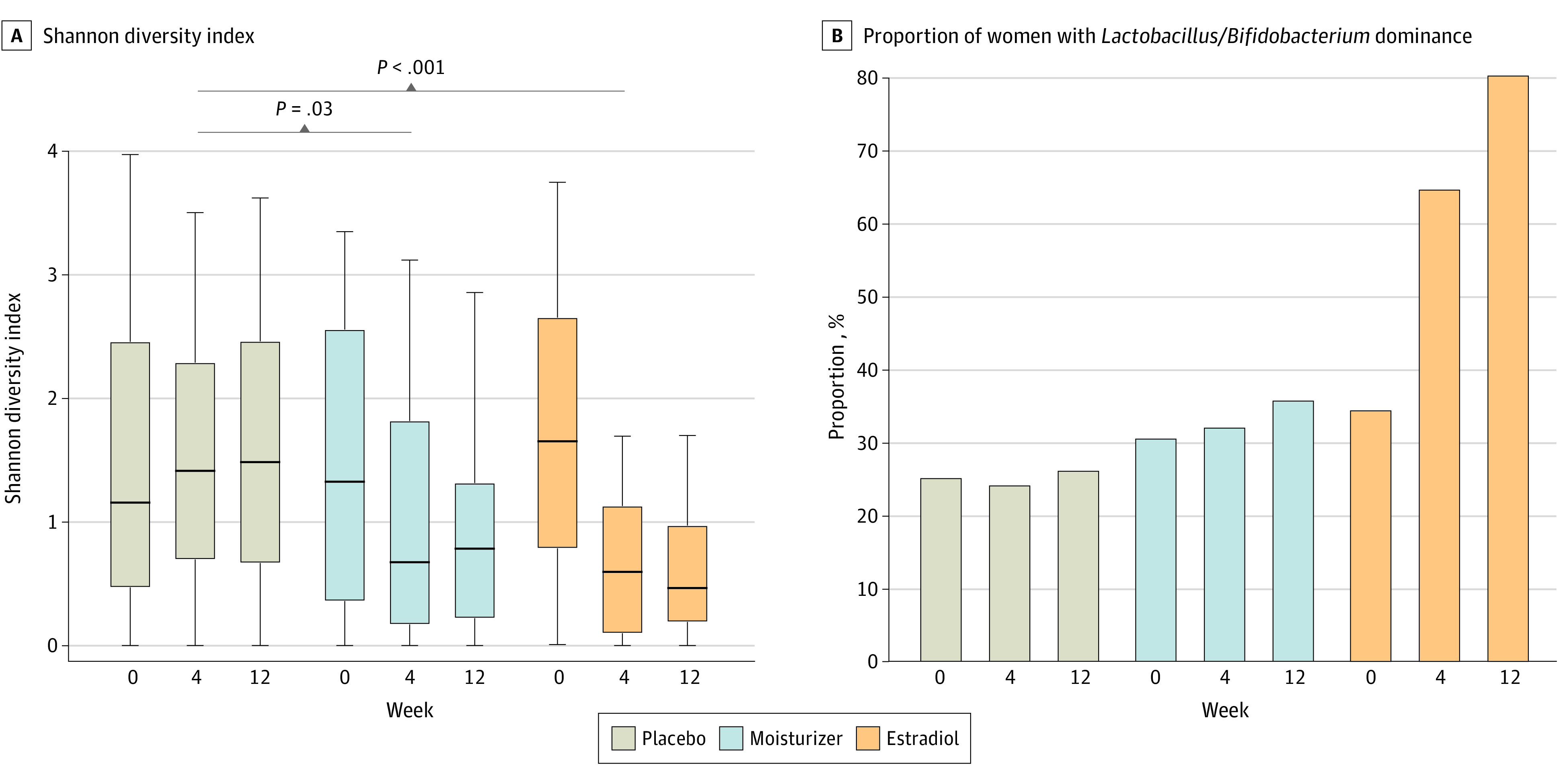
Changes in the Vaginal Microbiota With Estradiol, Moisturizer, or Placebo Use

**Figure 2.**
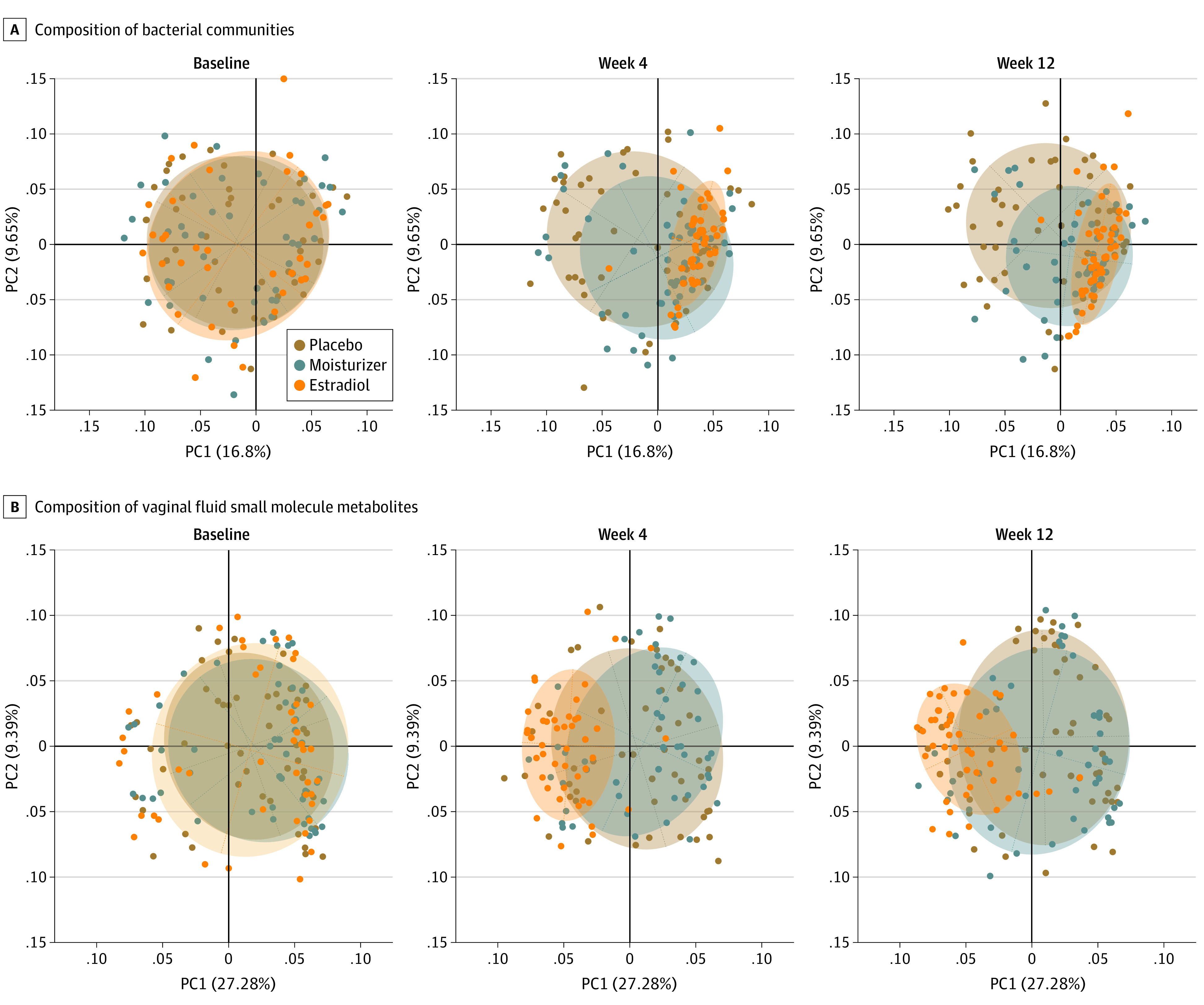
β Diversity Plots Depicting Shifts in the Vaginal Microbiota and Metabolome With Estradiol, Moisturizer, or Placebo Use Each dot represents the vaginal bacterial community or metabolic profile in a single participant. Overlapping circles indicate no differences between the groups. By week 4, women in the estradiol group had different vaginal bacterial communities (*P* < .001) and metabolic profiles (*P* < .001) compared with women in the placebo group. These changes were maintained at week 12. Such significant shifts in the microbiota (*P* = .08) or metabolic profiles was not observed among women in the moisturizer group (*P* = .72). PC indicates principal coordinates.

Median (IQR) SDI at 12 weeks showed decreases in both estradiol (baseline: 1.7 [0.8-2.7]; 12 weeks: 0.5 [0.2-1.0]; *P* < .001) and moisturizer (baseline: 1.3 [0.4-2.6]; 12 weeks: 0.8 [0.2-1.3]; *P* = .03) groups compared with the placebo group (baseline: 1.2 [0.5-2.5]; 12 weeks: 1.5 [0.7-2.5]) (Figure 1A). At 12 weeks, 36 women (80%) in the estradiol group had bacterial communities dominated by *Lactobacillus* and *Bifidobacterium,* compared with 13 women (26%) in the placebo group (*P* < .001) (Figure 1B; eFigure 1 in Supplement 2). Analyses of β diversity showed significant shifts in composition of bacterial communities by 12 weeks among women in the estradiol group compared with the placebo group (*P* < .001) but not moisturizer vs placebo groups (*P* = .08) (Figure 2A). Relative abundances of 31 individual bacterial taxa changed after 12 weeks of estradiol treatment (eTable 1 in Supplement 2). Among the top 10 significant taxa, abundances of lactobacilli and bifidobacteria were higher, while abundances of *Streptococcus mitis*, *Peptoniphilus* spp, *Anaerococcus vaginalis,* and *Finegoldia magna* decreased from baseline with estradiol use (eTable 1 in Supplement 2). In contrast, abundances of 11 bacterial taxa were significantly changed at 12 weeks among women in the moisturizer group, including increase in *Lactobacillus acidophilus,* or *Lactobacillus kitasatonis* and decreases in *S mitis*, *Prevotella timonensis*, *Peptoniphilus coxii,* and *A vaginalis* (eTable 2 in Supplement 2). We did not note increases in abundance of other lactobacilli or bifidobacteria in the moisturizer group (eTable 2 in Supplement 2). Instead, there was an increase in *Enterococcus faecalis* (eTable 2 in Supplement 2), which was not observed with estradiol use (eTable 1 in Supplement 2).

### Impact of Moisturizer and Estradiol Treatment on the Vaginal Metabolome and pH

**Figure 3.**
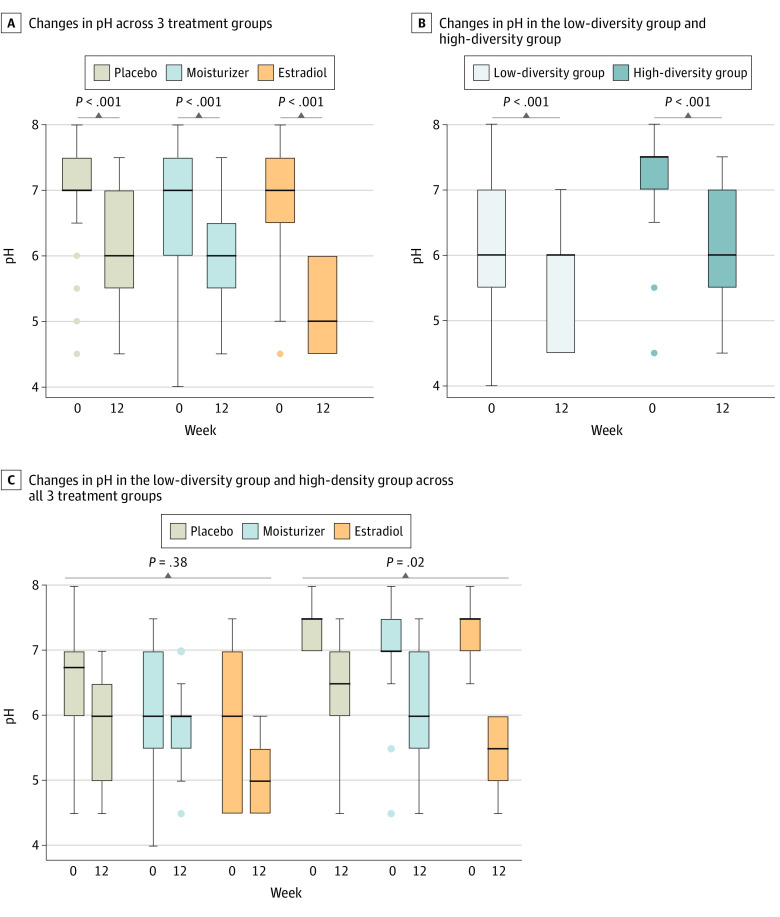
Vaginal pH Among Women Using Estradiol, Moisturizer, or Placebo Lines indicate medians; boxes, IQRs; error bars, ranges.

The composition of vaginal fluid small molecule metabolites significantly changed from baseline to 12 weeks with estradiol treatment (*P* < .001) but not with moisturizer use (*P* = .72) (Figure 2B). Of 171 metabolites measured, 90 (53%), from multiple metabolic pathways, were significantly changed in women using estradiol vs placebo: 41 metabolites were higher and 49 metabolites were lower (eTable 3 in Supplement 2). We noted significant increases in abundances of lactate, indole-3-lactate, and phenyl-lactate metabolites in the estradiol group vs placebo group, which can contribute to low pH (eTable 3 in Supplement 2), but which were not observed in the moisturizer vs placebo groups (eTable 4 in Supplement 2). Consistent with shifts in metabolites, vaginal pH at 12 weeks among women using estradiol was significantly lower vs the placebo group (median [IQR] pH, 5 [4.5-6.0] vs 6 [5.5-7.0]; *P* = .005), but there was no statistically significant difference for women in the moisturizer group (median [IQR] pH, 6 [5.5-6.5]; *P* = .28). However, pH significantly decreased over 12 weeks within each treatment group, reflecting the low pH formulations of both the moisturizer and the placebo (Figure 3A).

### Association Between Changes in the Vaginal Microbiota and Metabolites

Given the consistent shifts across 12 weeks with estradiol use, we examined the association between changes in the microbiota and small molecule metabolites. Hierarchical clustering of the vaginal microbiota at baseline identified 2 subgroups of study participants: 1 subgroup of women had vaginal microbiotas with lower SDI compared with women in the second subgroup (median [IQR] SDI, 0.6 [0.1-1.1] vs 2.5 [1.7-3.0]; *P* < .001) (eFigure 2 in Supplement 2). Similarly, the metabolites at baseline largely separated according to the microbiota groups noted previously (eFigure 3 in Supplement 2). The median pH at baseline of the low-diversity subgroup was lower than the pH of the high-diversity subgroup, and those participants were more likely to have more than 5% superficial cells on VMI (eTable 5 in Supplement 2). Among 6 Black women included in our analysis, all were in the low-diversity subgroup. Overall, the microbiota and metabolites were highly correlated at baseline (eFigure 4 in Supplement 2). Furthermore, α diversity and pH were highly correlated: samples with low SDI also had lower pH (eFigure 4 in Supplement 2). Median change in pH was significantly greater over 12 weeks for women in the high-diversity subgroup compared with the low-diversity subgroup (median [IQR] pH, −1.0 [−2.0 to −0.5] vs −0.3 [−1.1 to 0]; *P* = .007); however, the reduction in pH over 12 weeks was significant within both diversity subgroups (Figure 3B). Decreases in pH were observed in women in the high-diversity subgroup across all 3 intervention groups (Figure 3C). Moreover, similar proportions of women in the high- vs low-diversity subgroups had a shift to more than 5% superficial cells on VMI (15 of 49 women [31%] vs 12 of 45 women [27%]).

**Figure 4.**
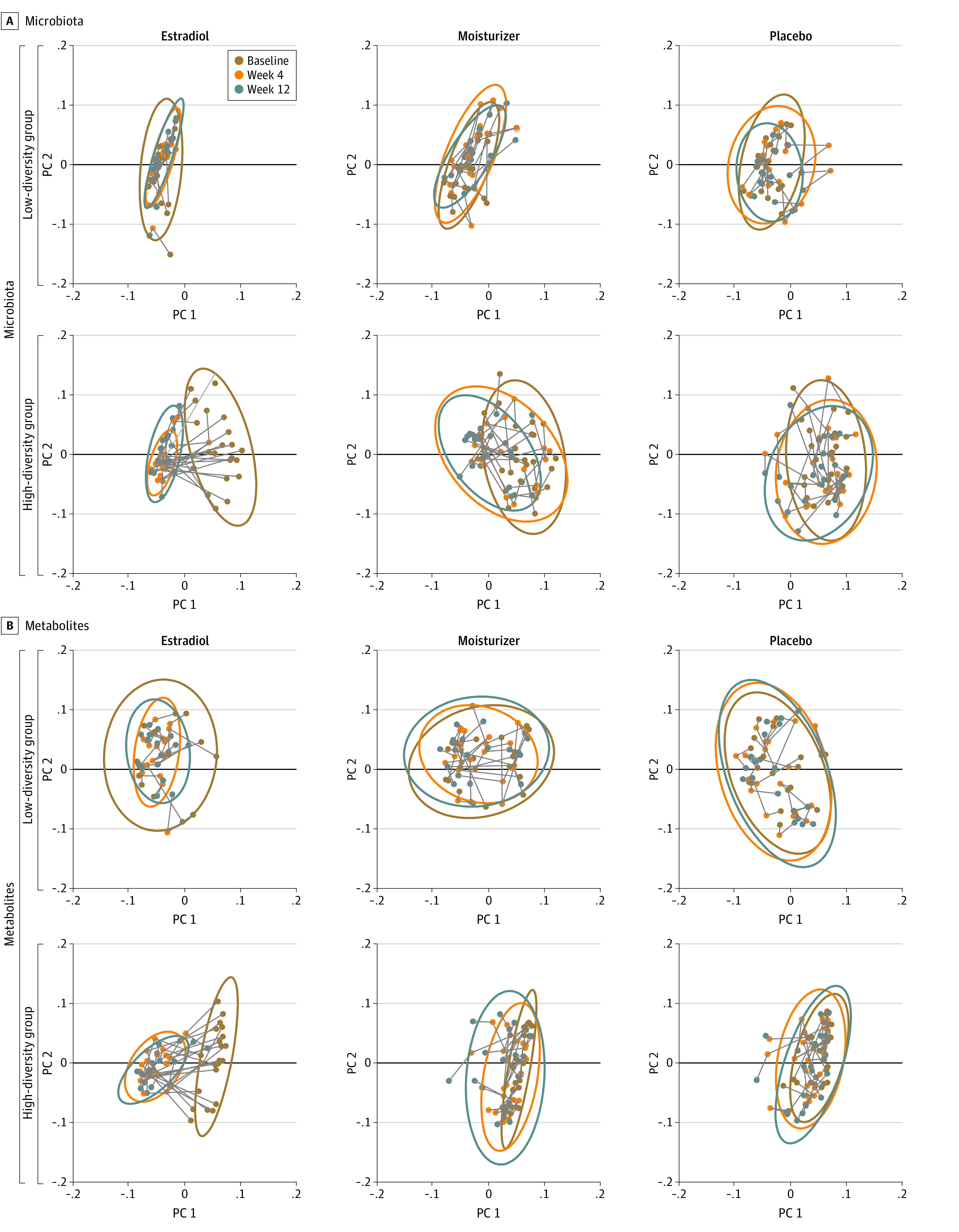
Metabolite and Microbiota Shifts Among Women in the High- and Low-Diversity Subgroups Each dot represents the vaginal bacterial community (A) or metabolic profile (B) in a single study participant and by time in study. PC indicates principal coordinates.

We noted strong positive correlations between changes in the microbiota and changes in metabolites across 12 weeks among women using estradiol; maximal changes occurred with 4 weeks of estradiol use (eFigure 4 in Supplement 2). Importantly, there was an orchestrated shift in the microbiota (Figure 4A) and metabolites (Figure 4B) with estradiol use from the high-diversity subgroup toward the low-diversity subgroup, while such concerted shifts were not noted with moisturizer or placebo use. Women in the low-diversity subgroup maintained these communities and metabolites across all 3 intervention groups over 12 weeks (Figure 4). Moreover, there was no difference in the proportion of women with a decrease of 2 points or more in symptom severity between high- vs low-diversity baseline subgroups (eTable 5 in Supplement 2).

### Impact of Serum Estradiol Levels on the Vaginal Microbiota and pH

We measured serum estradiol concentrations in the estradiol and placebo groups to determine whether overall differences or changes noted in the vaginal microenvironment were a response to circulating estradiol. We noted a significant increase in serum estradiol concentrations with estradiol treatment (median [IQR] estradiol, 4.0 [3.1-5.4] pg/mL at 12 weeks vs 3.6 [2.6-4.5] pg/mL at baseline; *P* = .02) but not placebo (median [IQR] estradiol, 3.1 [2.2-4.4] pg/mL at 12 weeks vs 3.1 [2.1-4.7] pg/mL at baseline; *P* = .48) (eFigure 5 in Supplement 2). No changes were noted across 12 weeks in estradiol concentrations in the low- or high-diversity subgroups (eFigure 5 in Supplement 2). Estradiol levels at baseline also did not vary significantly between estradiol and placebo groups (median [IQR] 3.6 [3.1-4.7] pg/mL vs 3.1 [2.1-4.7] pg/mL; *P* = .14), nor did estradiol levels vary between high- vs low-diversity subgroups at baseline (median [IQR], 3.6 [2.6-4.7] pg/mL vs 3.1 [2.3-4.2] pg/mL; *P* = .23) or 12 weeks (median [IQR], 3.4 [2.7-4.8] pg/mL vs 3.4 [2.6-5.0] pg/mL, *P* = .73). Interestingly, increase in serum estrogen concentration between week 12 compared with baseline (median [IQR] increase, 0.4 [−0.3 to 1.2] pg/mL) was not associated with change in SDI (median [IQR] change, –1.31 [–1.96 to –0.06]; *P* = .71) or pH (median [IQR] change, –1.5 [–2.0 to –1.0]; *P* = .27) among women using estradiol, despite significant decreases in SDI and pH in this group, suggesting that local concentrations of estradiol may play a greater role than systemic concentrations in mediating changes in the vagina.

## Discussion

In this post hoc secondary analysis of participants in a randomized clinical trial of topical treatment for moderate to severe postmenopausal vaginal symptoms, we demonstrated a significantly greater impact of vaginal estradiol on the vaginal microbiota, metabolome, and pH than low-pH vaginal moisturizer or low-pH placebo gel. Although participants in all treatment groups had significant decreases in vaginal pH over 12 weeks, participants in the estradiol group had the largest decrease, associated with changes in the composition of the vaginal microbiota and metabolites. The effect of estradiol was greatest in women with a high-diversity vaginal microbial community, high pH, and low VMI at study entry and was not associated with changes in serum levels of estrogen.

Many treatment trials of postmenopausal vaginal discomfort measure clinician-driven metrics, such as visual appearance on examination, pH, or vaginal epithelial cell maturation.^16,17^ In a meta-analysis of randomized trials, vaginal estradiol was associated with significant improvement in these findings to a greater extent than placebo, but not necessarily women’s report of symptoms.^3^ Our in-depth analysis demonstrates the same disconnect: estradiol was associated with significant, profound changes in microbiota, small molecule metabolites, and pH of the vagina but did not confer a significantly greater symptom benefit than placebo.^1^ The placebo gel had high lubricity, which likely conferred the symptom benefit without microbiota and metabolite changes in the vaginal microenvironment.

Change in pH is 1 of 3 FDA-recommended primary outcomes for treatment trials in GSM. A significant decrease in vaginal pH has been seen in trials of vaginal estrogen,^3,18^ vaginal DHEA,^19^ vaginal laser,^20^ hyaluronic acid, and vaginal gels containing lactic acid.^2,21,22^ Our findings demonstrate that a significant drop in pH with treatment does not necessarily reflect the same underlying biological effect in all cases. In premenopausal women, pH is often linked to levels of vaginal lactic acid. The estradiol group alone had significant changes in metabolic features that contribute to low pH compared with placebo. In the popular press, advertising language touts “pH-balanced” products for women, and there have been many attempts to promote vaginal health by decreasing pH alone.^23,24,25,26^ However, none of the low-pH products studied in premenopausal women have decreased incidence of BV.^27,28^ In fact, a 2019 study by van der Veer et al^26^ found that use of 1 lactic acid–based douche in premenopausal women increased risk for having diverse communities of vaginal anaerobes. Our analysis shows that a decrease only in vaginal pH is insufficient to change the microbiota in postmenopausal women. Additionally, the metabolites in vaginal fluid did not shift significantly with low-pH interventions, even among women with high-diversity microbial communities, suggesting that lowering pH alone may not alter underlying microbial or host metabolic processes.

It is not surprising that participants in our trial with a high-diversity microbiota at enrollment had a greater change in diversity of the microbial community with vaginal estrogen. What is of interest is that vaginal estrogen contributed to an additional reduction in pH even among women with a low-diversity community, suggesting that estradiol facilitates an increase in metabolic activity of lactic acid–producers, such as lactobacilli and bifidobacteria. Moreover, even among women who started with high-diversity communities, there was no difference in the proportion with a large reduction in symptom severity (ie, ≥2 points) among treatment groups, confirming a lack of causal association between microbiota and postmenopausal vaginal symptom severity. In our original analysis, participants in the estradiol group were more likely to report meaningful benefit from the estradiol intervention than the other 2 groups. It is possible that the microbial and metabolic changes with estradiol use may contribute to overall well-being in ways not measured by the symptom questionnaire.

In premenopausal women, higher relative abundance of lactobacilli in the vagina is associated with lower levels of proinflammatory cytokines and chemokines.^29^ The inflammation associated with diverse microbes (and higher pH) is proposed as 1 contributor to increased risks for cervical dysplasia and HIV acquisition. While a few studies have demonstrated differences in vaginal fluid immune markers between premenopausal and postmenopausal women, the contribution of the vaginal microbiota or metabolites to these differences has not been evaluated.^30,31^ A small randomized clinical trial of a vaginal probiotic in postmenopausal women found short-term decreases in proinflammatory gene pathways with increased vaginal lactobacilli.^32^ Together, these data suggest that increasing vaginal lactobacilli in postmenopausal women may improve vaginal health regardless of impact on symptoms and should be an area of future investigation.

### Limitations

This study has some limitations. This is a subset analysis of a limited number of trial participants; hence, our findings may not be generalizable. However, we included participants who reported greatest adherence to study intervention and thus likely reflect biological effects of adherent use. Participants were selected to represent a range of response to treatment, providing additional breadth to our analysis. We only collected samples at 3 points during the trial, so a detailed time course of the kinetics of the noted changes is not possible. Additionally, we do not know if these changes are durable over longer periods of therapy, nor whether longer-term treatment might lead to additional or different shifts in microbes or metabolites. However, our data suggest that much of the change occurred within 4 weeks. The low-pH products used in our trial achieved low pH using sorbic acid and not lactic acid, and it is possible that the 2 acids might have different effects on microbial communities. As noted in our initial report,^1^ our trial participants mostly identified as White, limiting generalizability. The need to increase participant diversity in studies of postmenopausal women is highlighted by our finding that the 6 Black women in our analysis were all categorized in the low-diversity subgroup; data from premenopausal women suggest that Black women have diverse bacterial communities.^9,33^

## Conclusions

The findings of this secondary analysis of a randomized clinical trial suggest that a significant decrease in pH over the course of a trial may not reflect the same underlying biological processes among different interventions, and thus, lowering pH should not be a primary goal. The metric of lowering of vaginal pH per FDA guidance for primary efficacy analyses in clinical trials in GSM is a blunt measure.^7^ In the era of precision medicine, trial outcomes should reflect patient-reported outcomes and specific biological effects. The MsFLASH Vaginal Health Trial demonstrated that although the biological effects of estrogen on the vaginal microenvironment may not be linked to symptom improvement, they may reflect an important change in vaginal mucosal characteristics not observed with products that simply lower pH.

## Notes

Supplement 1.
Trial Protocol and Statistical Analysis Plan

Supplement 2.
eMethods.
**eFigure 1.** Relative Abundance and α Diversity in the Placebo, Moisturizer and Estradiol Groups at Baseline, Week 4 and Week 12 **eFigure 2.** Unsupervised Hierarchical Clustering of Bacterial Communities at Baseline **eFigure 3.** Unsupervised Hierarchical Clustering of Metabolic Profiles at Baseline **eFigure 4.** Vaginal Microbiota and Metabolites are Highly Correlated **eFigure 5.** Small but Significant Increase in Serum Estradiol Concentrations of Women Using Vaginal Estradiol **eTable 1.** Comparison of Change in Individual Bacterial Taxa in the Estradiol Group vs the Placebo Group **eTable 2.** Comparison of Change in Individual Bacterial Taxa in the Moisturizer vs the Placebo Group **eTable 3.** Comparison of Change in Individual Metabolites in the Estradiol Group vs the Placebo Group **eTable 4.** Comparison of Change in Individual Metabolites in the Moisturizer Group vs the Placebo Group **eTable 5. **Demographic and Clinical Characteristics of Participants by Microbial Diversity Grouping at Baseline

Supplement 3.
Data Sharing Statement
