# Supplementary material for: Impact of Topical Interventions on the Vaginal Microbiota and Metabolome in Postmenopausal Women: A Secondary Analysis of a Randomized Clinical Trial
Source: JAMA Netw Open. 2022 Mar 30;5(3):e225032. doi: 10.1001/jamanetworkopen.2022.5032 (PMC8968546; doi:10.1001/jamanetworkopen.2022.5032)
Supplement: Supplement 2. — eMethods. eFigure 1. Relative Abundance and α Diversity in the Placebo, Moisturizer and Estradiol Groups at Baseline, Week 4 and Week 12 eFigure 2. Unsupervised Hierarchical Clustering of Bacterial Communities at Baseline eFigure 3. Unsupervised Hierarchical Clustering of Metabolic Profiles at Baseline eFigure 4. Vaginal Microbiota and Metabolites are Highly Correlated eFigure 5. Small but Significant Increase in Serum Estradiol Concentrations of Women Using Vaginal Estradiol eTable 1. Comparison of Change in Individual Bacterial Taxa in the Estradiol Group vs the Placebo Group eTable 2. Comparison of Change in Individual Bacterial Taxa in the Moisturizer vs the Placebo Group eTable 3. Comparison of Change in Individual Metabolites in the Estradiol Group vs the Placebo Group eTable 4. Comparison of Change in Individual Metabolites in the Moisturizer Group vs the Placebo Group eTable 5. Demographic and Clinical Characteristics of Participants by Microbial Diversity Grouping at Baseline [file jamanetwopen-e225032-s002.pdf]

Supplemental Online Content

Srinivasan S, Hua X, Wu MC, et al. Impact of topical interventions on the vaginal microbiota and metabolome in postmenopausal women: a secondary analysis of a randomized clinical trial. *JAMA Netw Open*. 2022;5(3):e225032.
doi:10.1001/jamanetworkopen.2022.5032

eMethods.

eFigure 1. Relative Abundance and α Diversity in the Placebo, Moisturizer and Estradiol Groups at Baseline, Week 4 and Week 12

eFigure 2. Unsupervised Hierarchical Clustering of Bacterial Communities at Baseline

eFigure 3. Unsupervised Hierarchical Clustering of Metabolic Profiles at Baseline

eFigure 4. Vaginal Microbiota and Metabolites are Highly Correlated

eFigure 5. Small but Significant Increase in Serum Estradiol Concentrations of Women Using Vaginal Estradiol

eTable 1. Comparison of Change in Individual Bacterial Taxa in the Estradiol Group vs the Placebo Group

eTable 2. Comparison of Change in Individual Bacterial Taxa in the Moisturizer vs the Placebo Group

eTable 3. Comparison of Change in Individual Metabolites in the Estradiol Group vs the Placebo Group

eTable 4. Comparison of Change in Individual Metabolites in the Moisturizer Group vs the Placebo Group

eTable 5. Demographic and Clinical Characteristics of Participants by Microbial Diversity Grouping at Baseline

This supplemental material has been provided by the authors to give readers additional information about their work.

© 2022 Srinivasan S et al. *JAMA Network Open.*

eMethods.

Molecular Methods: DNA from vaginal swabs was extracted using the BiOstic Bacteremia kit (Qiagen, Germantown, MD). Blank swabs (negative controls) were extracted to monitor potential laboratory contamination. PCR inhibition was evaluated using an internal amplification control assay.[1] Bacterial DNA concentrations were measured using a TaqMan-based qPCR assay.[2] The vaginal microbiota was characterized by sequencing on the Illumina MiSeq instrument (Illumina, San Diego, CA).[3,4] The *DADA2* package was used for processing reads resulting in a list of unique sequence variants (SVs); taxonomy was assigned to the SVs by placing on a custom phylogenetic tree.[2,3,4] Sequences have been deposited in the NCBI SRA (PRJNA788936).

Metabolomic Profiling: Broad-based metabolomics was performed on a liquid-chromatography-mass spectrometry (LC-MS/MS) platform at Northwest Metabolomics Research Center.[5] Aqueous metabolites were extracted from vaginal fluid samples using methanol, the supernatant was dried and reconstituted in solvents containing isotope-labeled internal standards to monitor LC-MS assay performance. Pooled study samples and commercially available pooled serum (Innovative Research, Novi, MI) were extracted in the same manner as samples and used as quality controls (QCs) to monitor instrument performance and batch-to-batch normalization. Samples were analyzed with targeted LC-MS/MS based on an AB SCIEX 6500 triple quad mass spectrometer (Framingham, MA) using negative- and positive-ionization modes. Pooled study QC samples were run before and after every 10 samples to monitor instrument performance.

Estradiol concentrations in serum samples: Serum estradiol levels were measured at the Brigham Research Assay Core Laboratory (Boston, MA) using LC-MS/MS on the AB-SCIEX Triple Quad 5500+ system post-derivatization with dansyl chloride and addition of deuterated

© 2022 Srinivasan S et al. *JAMA Network Open.*

estradiol to each sample as an internal standard.[6,7] The linear range of the assay was 1-500 pg/mL; lower limit of quantitation, 1 pg/mL. Inter-assay CVs were 6.9%, 7.0%, 4.8% at concentrations of 8, 77, 206 pg/mL, respectively. Cross-reactivity of estrone, testosterone, DHEA, DHEAS, DHT, androstenedione was negligible at ten times the circulating concentrations of these hormones. This assay is certified by CDC's Hormone Standardization Program; mean bias for QC samples provided by CDC Hormone Standardization Program was 0.81 pg/mL for estradiol concentrations ≤20 pg/mL, and 1.9% estradiol concentrations >20 pg/mL.

References

1. Khot PD, Ko DL, Hackman RC, Fredricks DN. Development and optimization of quantitative PCR for the diagnosis of invasive aspergillosis with bronchoalveolar lavage fluid. *BMC infectious diseases.* 2008;8:73.
2. Srinivasan S, Hoffman NG, Morgan MT, et al. Bacterial communities in women with bacterial vaginosis: high resolution phylogenetic analyses reveal relationships of microbiota to clinical criteria. *PLoS One.* 2012;7(6):e37818.
3. Golob JL, Pergam SA, Srinivasan S, et al. Stool Microbiota at Neutrophil Recovery Is Predictive for Severe Acute Graft vs Host Disease After Hematopoietic Cell Transplantation. *Clinical infectious diseases : an official publication of the Infectious Diseases Society of America.* 2017;65(12):1984-1991.
4. Srinivasan S, Chambers LC, Tapia KA, et al. Urethral Microbiota in Men: Association of Haemophilus influenzae and Mycoplasma penetrans With Nongonococcal Urethritis. *Clin Infect Dis.* 2021;73(7):e1684-e1693.
5. Srinivasan S, Morgan MT, Fiedler TL, et al. Metabolic signatures of bacterial vaginosis. *mBio.* 2015;6(2).
6. Snyder PJ, Bhasin S, Cunningham GR, et al. Effects of Testosterone Treatment in Older Men. *N Engl J Med.* 2016;374(7):611-624.
7. Jasuja GK, Travison TG, Davda M, et al. Age trends in estradiol and estrone levels measured using liquid chromatography tandem mass spectrometry in community-dwelling men of the Framingham Heart Study. J Gerontol A Biol Sci Med Sci. 2013;68(6):733-740.

© 2022 Srinivasan S et al. *JAMA Network Open.*

FIGURE LEGENDS

eFigure 1: Relative abundance of bacterial taxa shown at the species or genus level for visualization. Bacterial taxa less than 1% abundance were categorized in the "other" group. Alpha diversity (Shannon Diversity Index) shown below the relative abundances for each participant at each timepoint.

eFigure 2: Unsupervised hierarchical clustering of vaginal bacterial communities at baseline separated women into two groups. One group had a lower SDI compared to women in the other group (0.60 vs 2.51, $P<0.001$), hence labeled as Low Diversity and High Diversity Groups.

eFigure 3: Unsupervised hierarchical clustering of metabolic profiles at baseline separated women largely into their baseline diversity groups suggesting a correlation between the vaginal microbiota and small molecule metabolites.

eFigure 4: The vaginal microbiota and small molecule metabolites are correlated as depicted in the principal coordinates plot showing the top principal coordinate for each data element (**A**). Dots are colored by diversity group at baseline (See eFigure 3). A Locally Weighted Scatterplot Smoothing (LOWESS) line (red, dashed) was added to help visualize the correlation between the microbiota and metabolite profiles. The pH of samples at baseline was also highly corelated with alpha diversity (Shannon Diversity Index) (**B**). A consistent positive correlation between the microbiota and metabolites were noted over time, with the strongest correlation noted among estradiol users (**C**). LOWESS lines, colored by intervention arm, was added to each panel to help visualize the correlation between the microbiota and metabolites. Maximal changes in the composition of the microbiota and metabolites occurred at the 4-week timepoint and changes between week 4 and week 12 were relatively small (**C**).

eFigure 5: Use of 10-µg estradiol tablet (plus placebo gel) nightly for 2-weeks and then 2-times for 2-weeks resulted in a small but significant increase in serum estradiol concentrations

© 2022 Srinivasan S et al. *JAMA Network Open.*

(P=0.015) (**5A**). Such a change was not noted among the placebo group (P=0.48). There were no changes in serum estradiol concentrations by week 12 among women in the Low (P=0.14) or High Diversity (P=0.91) groups (**5B**).

© 2022 Srinivasan S et al. *JAMA Network Open.*

Supplement 2, eFigure 1

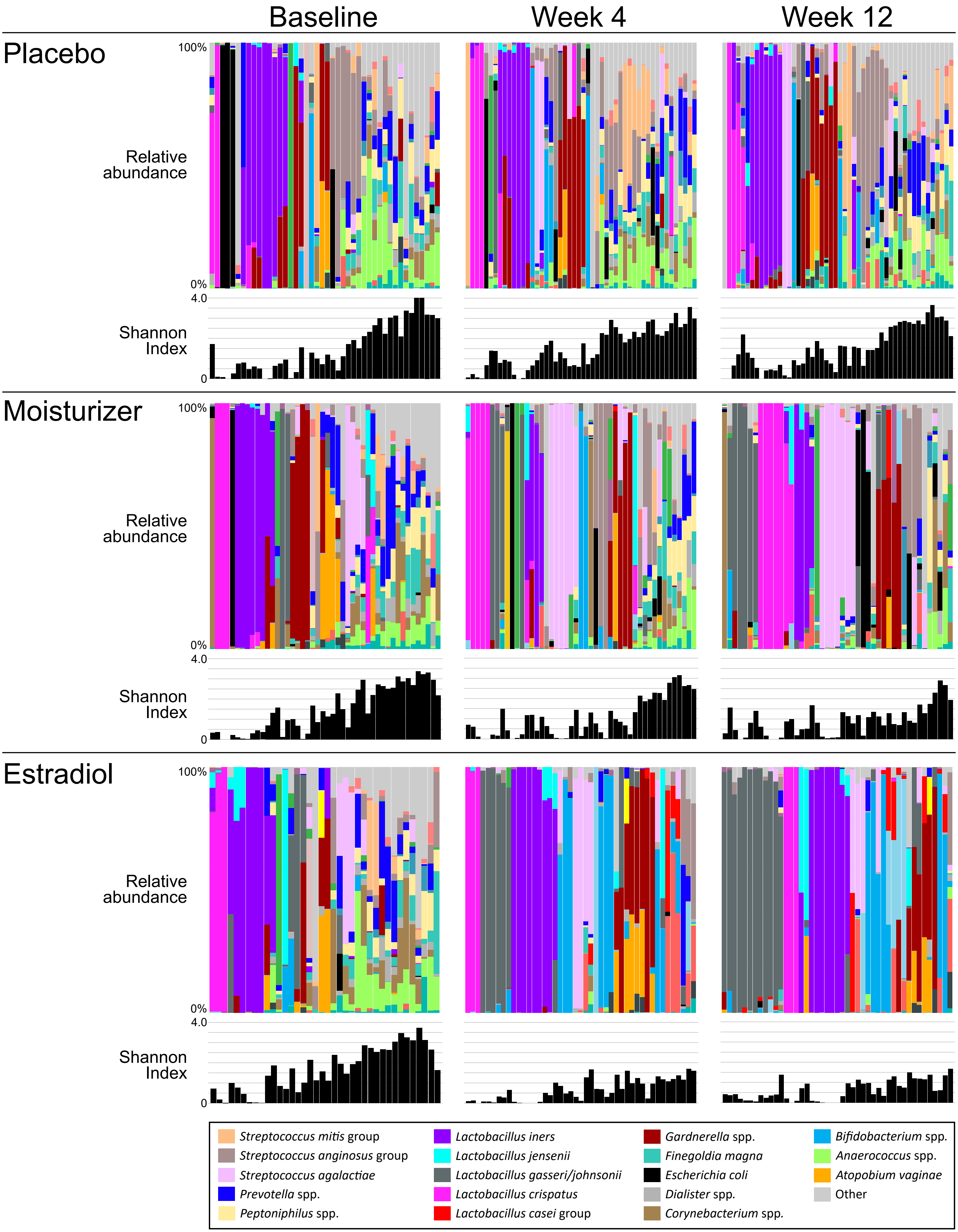

© 2022 Srinivasan S et al. JAMA Network Open.

Supplement 2, eFigure 2

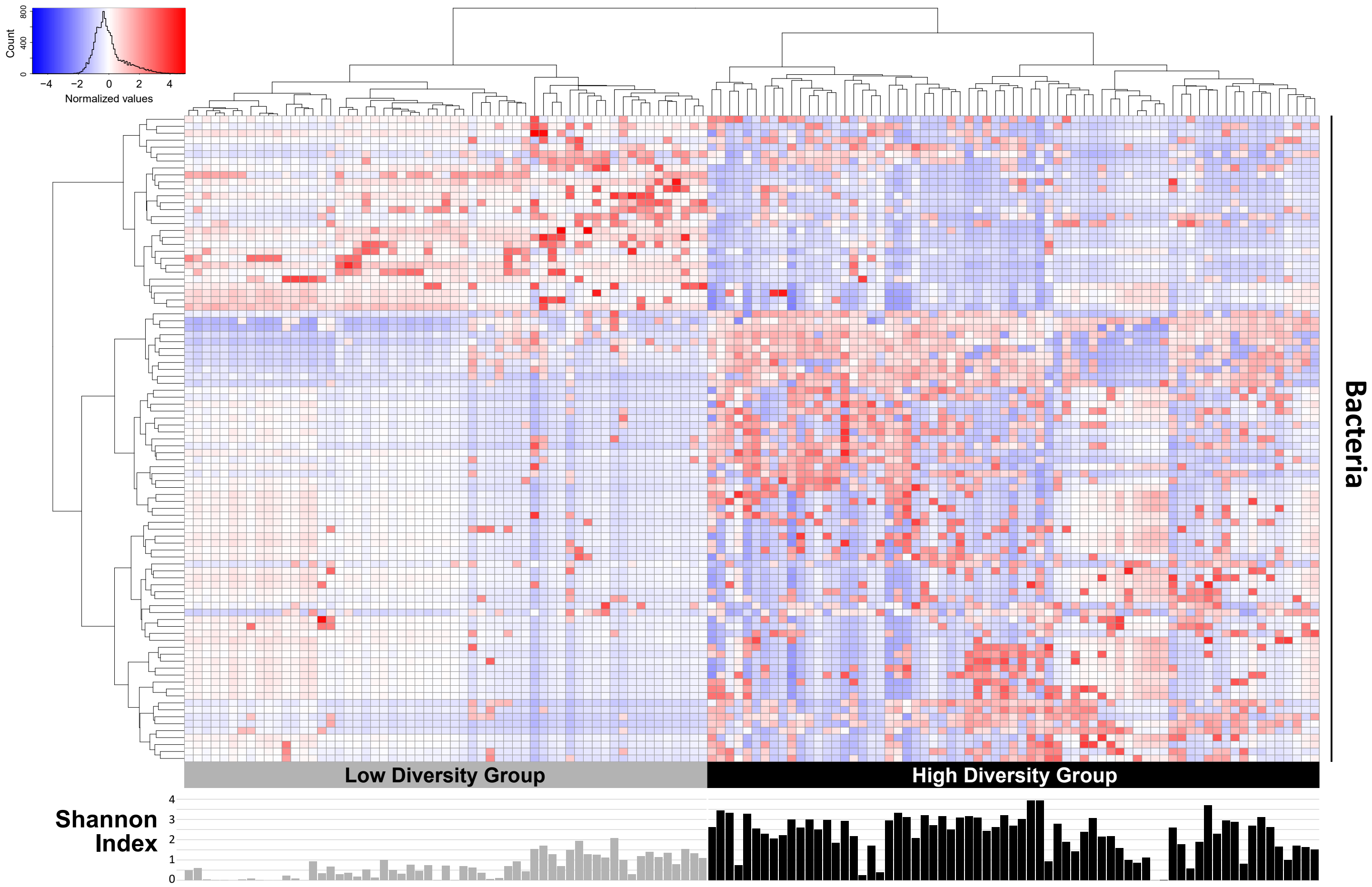

© 2022 Srinivasan S et al. JAMA Network Open.

Supplement 2, eFigure 3

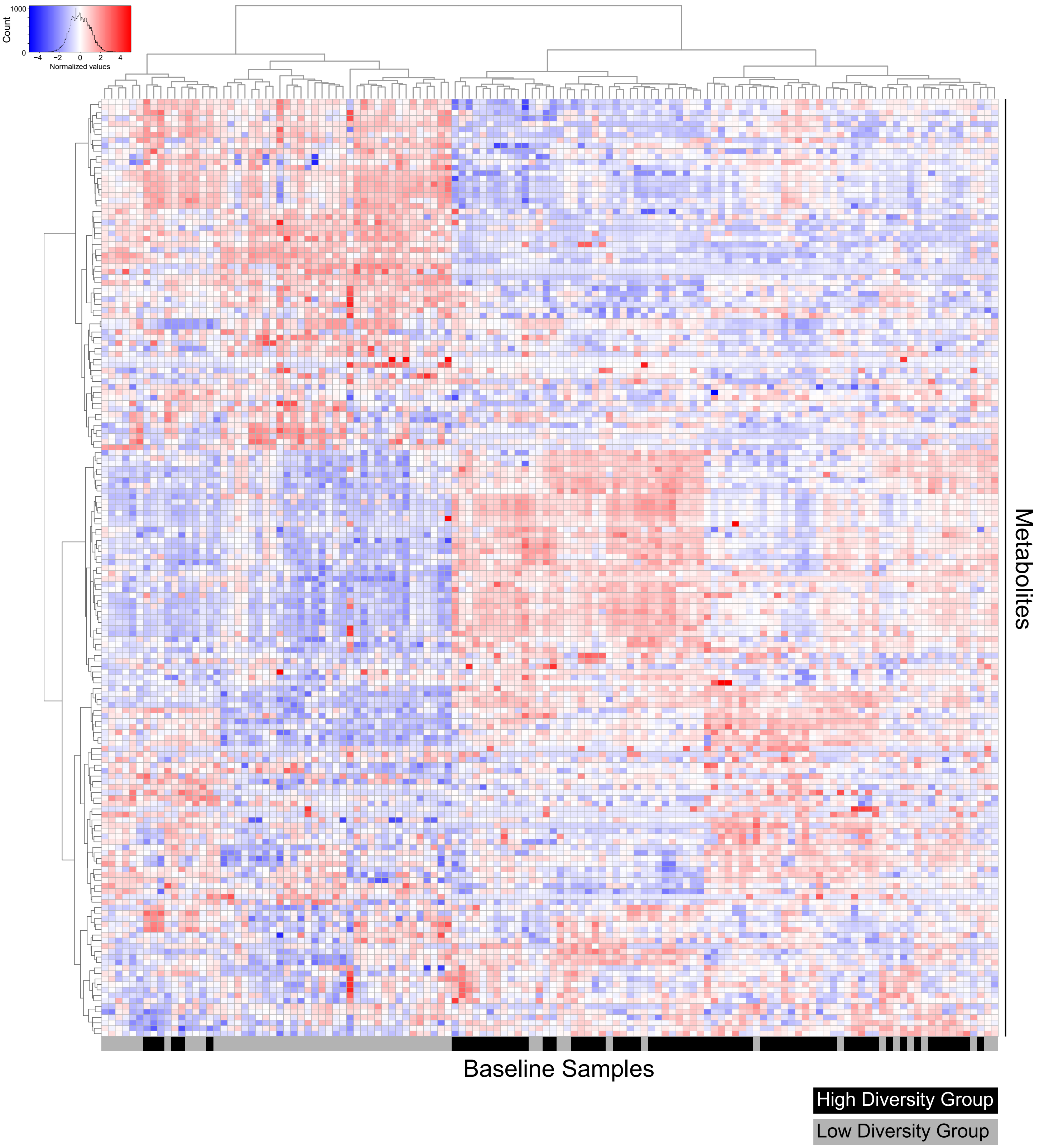

© 2022 Srinivasan S et al. JAMA Network Open.

Supplement 2, eFigure 4

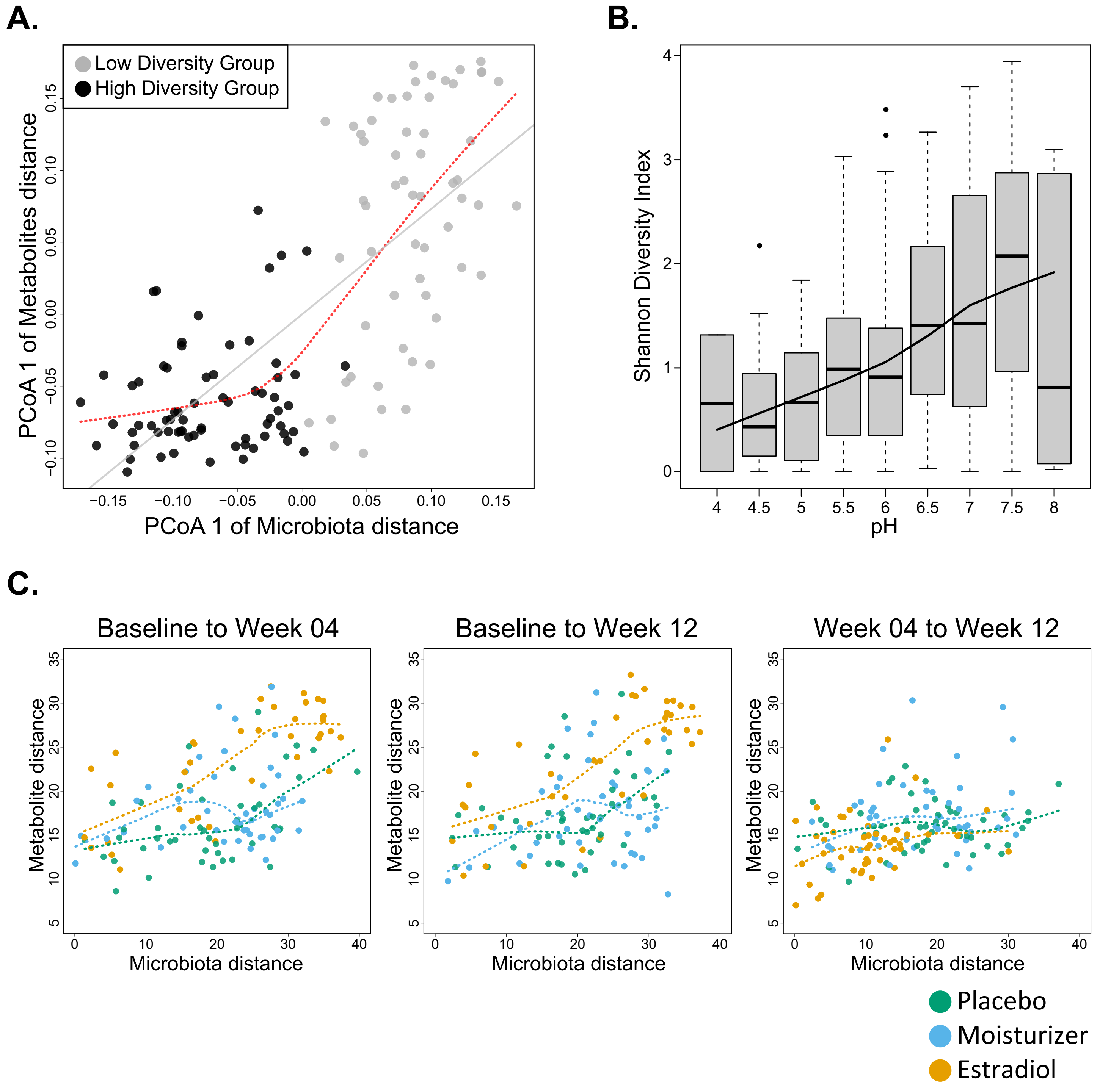

© 2022 Srinivasan S et al. JAMA Network Open.

Supplement 2, eFigure 5A

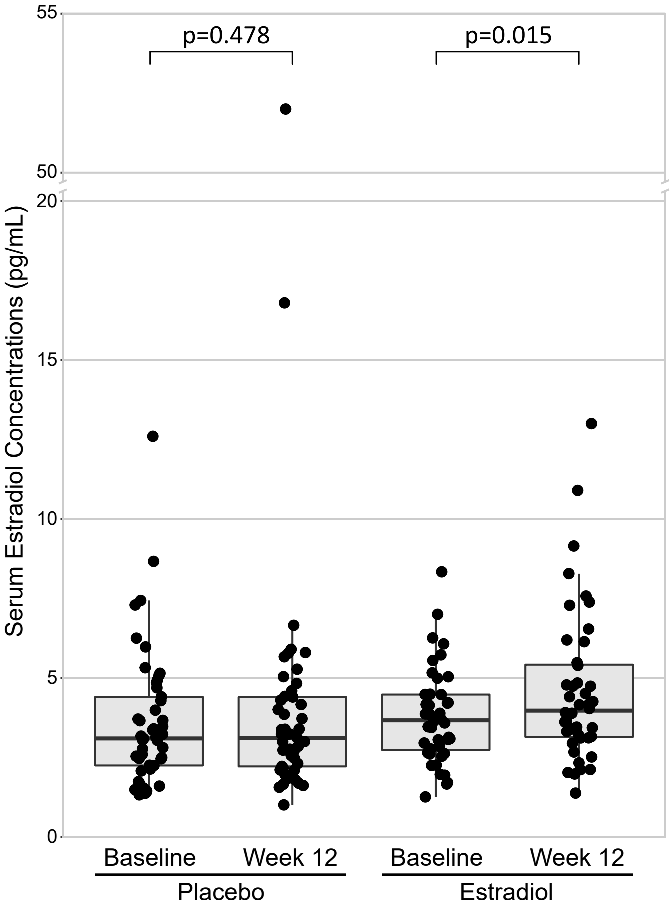

Supplement 2, eFigure 5B

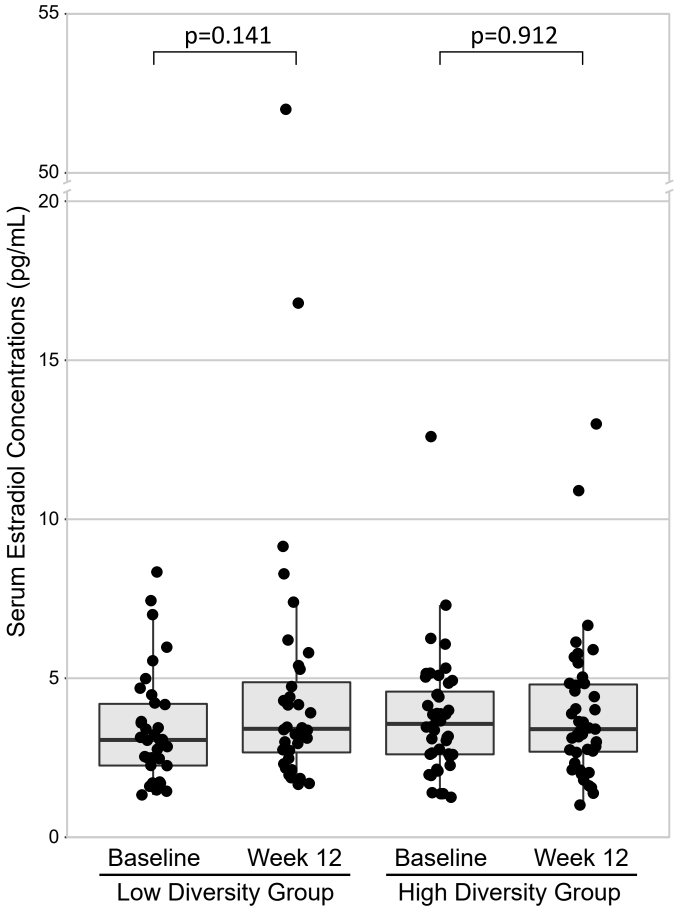

© 2022 Srinivasan S et al. JAMA Network Open.

Supplement 2, eTable 1: Comparison of change in individual bacterial taxa in the estradiol group versus the placebo group

Taxon	Estradiol (Est)	Estradiol (SE)	Estradiol (p-value)	Estradiol (q-value)
Lactobacillus casei group	1.688	0.286	<0.001	<0.001
Lactobacillus acidophilus/kitasatonis	1.269	0.292	<0.001	0.001
Streptococcus mitis group	-1.961	0.475	<0.001	0.002
Bifidobacterium bifidum	1.253	0.307	<0.001	0.002
Lactobacillus gasseri/johnsonii	2.271	0.567	<0.001	0.002
Peptoniphilus grossensis/harei/phoceensis	-1.591	0.402	<0.001	0.002
Anaerococcus vaginalis	-1.616	0.416	<0.001	0.002
Peptoniphilus coxii	-1.065	0.290	<0.001	0.004
Finegoldia magna	-1.488	0.405	<0.001	0.004
Bifidobacterium dentium	1.314	0.373	<0.001	0.005
Prevotella colorans	0.704	0.226	0.002	0.019
Prevotella timonensis	-1.261	0.448	0.006	0.044
Bifidobacterium breve	0.882	0.330	0.008	0.055
Corynebacterium pyruviciproducens	-0.740	0.281	0.009	0.055
Campylobacter ureolyticus	-0.741	0.286	0.010	0.055
Dialister propionicifaciens	-0.933	0.362	0.011	0.055
Streptococcus agalactiae	1.477	0.573	0.011	0.055
Anaerococcus mediterraneensis	-0.986	0.383	0.011	0.055
Corynebacterium genitalium	0.598	0.233	0.011	0.055
Varibaculum anthropi/cambriense	-0.968	0.384	0.013	0.060
Atopobium vaginae	1.225	0.500	0.015	0.068
Aerococcus christensenii	0.806	0.333	0.017	0.071
Lawsonella	-0.740	0.310	0.018	0.074
Lactobacillus jensenii	1.201	0.519	0.022	0.086
Streptococcus anginosus group	-1.401	0.620	0.025	0.094
Corynebacterium riegelii	0.556	0.248	0.027	0.096
Alloscardovia omnicolens	0.906	0.409	0.028	0.098
Howardella	0.836	0.385	0.032	0.105
Prevotella bivia	0.745	0.349	0.035	0.111
Bifidobacterium longum	0.550	0.276	0.048	0.147
Facklamia hominis	-0.603	0.304	0.049	0.147
Ezakiella	-0.662	0.341	0.054	0.156
Dialister micraerophilus	0.702	0.365	0.057	0.160
Facklamia ignava	0.459	0.242	0.059	0.163
Peptoniphilus	-0.634	0.337	0.062	0.165
Porphyromonas bennonis	-0.427	0.239	0.075	0.191
Mobiluncus curtisii	-0.483	0.270	0.076	0.191
Prevotella buccalis	-0.422	0.242	0.083	0.204
Actinomyces europaeus	0.411	0.244	0.094	0.224
Prevotella bergensis	-0.414	0.250	0.100	0.231
Gemella haemolysans/sanguinis	-0.461	0.287	0.111	0.251
Corynebacterium coyleae	0.405	0.262	0.125	0.277
Peptoniphilus lacrimalis	-0.372	0.248	0.136	0.295
Lactobacillus reuteri/vaginalis	0.561	0.384	0.146	0.308
Prevotella disiens	-0.578	0.400	0.150	0.311
Casaltella massiliensis	-0.341	0.251	0.177	0.353
Prevotella corporis	0.422	0.314	0.181	0.353
Corynebacterium pseudogenitalium/tuberculostearicum	-0.442	0.329	0.182	0.353
Fusobacterium nucleatum	-0.326	0.263	0.216	0.409
Porphyromonas uenonis	-0.355	0.289	0.220	0.409
Lactobacillus iners	0.887	0.744	0.235	0.424
Peptoniphilus koenoeneniae	0.285	0.242	0.241	0.424
Actinomyces turicensis	-0.384	0.327	0.242	0.424
Peptostreptococcus anaerobius	-0.328	0.284	0.249	0.429
Faecalibacterium prausnitzii	-0.301	0.267	0.260	0.440
Helcococcus	0.277	0.256	0.279	0.464
Fenollaria massiliensis/timonensis	-0.306	0.287	0.288	0.469
Dialister sp. type 2	0.400	0.392	0.309	0.496
Enterococcus faecalis	0.328	0.333	0.327	0.515
Corynebacterium mycetoides	0.260	0.275	0.346	0.537
Atopobium deltae	-0.235	0.255	0.360	0.548
Corynebacterium sundsvallense	0.260	0.289	0.371	0.556
Anaerococcus prevotii/tetradius	-0.266	0.304	0.382	0.562
Anaerococcus senegalensis	-0.229	0.265	0.389	0.562
Anaerococcus	-0.265	0.309	0.393	0.562
Escherichia coli	-0.297	0.355	0.405	0.571
Gardnerella vaginalis	0.526	0.679	0.440	0.602
Porphyromonas asaccharolytica	-0.187	0.242	0.440	0.602
Fastidiosipila sanguinis	0.164	0.228	0.474	0.638
Parvimonas	0.183	0.262	0.486	0.646
Lactobacillus crispatus	0.437	0.639	0.495	0.649
Candidatus Peptoniphilus massiliensis	0.135	0.230	0.558	0.720
Anaerococcus hydrogenalis/rubeinfantis	-0.158	0.309	0.610	0.777

© 2022 Srinivasan S et al. JAMA Network Open.

Taxon	Estradiol (Est)	Estradiol (SE)	Estradiol (p-value)	Estradiol (q-value)
Murdochiella asaccharolytica/Levyella massiliensis	-0.105	0.238	0.660	0.829
Corynebacterium simulans/striatum	0.114	0.278	0.683	0.846
Fenollaria	-0.104	0.271	0.701	0.858
Actinobaculum massiliense	-0.099	0.299	0.741	0.888
Ureaplasma parvum/urealyticum	0.112	0.344	0.745	0.888
Arcanobacterium	-0.069	0.238	0.774	0.911
Staphylococcus haemolyticus	0.070	0.265	0.791	0.919
Porphyromonas somerae	0.056	0.253	0.824	0.946
Actinomyces neuii	-0.084	0.424	0.843	0.956
Lagierella	0.039	0.247	0.876	0.966
Varibaculum	0.032	0.216	0.882	0.966
Corynebacterium	0.045	0.335	0.894	0.966
Staphylococcus capitis/caprae/epidermidis	-0.041	0.308	0.894	0.966
Corynebacterium amycolatum	-0.031	0.329	0.925	0.989
Anaerococcus lactolyticus	-0.018	0.272	0.948	0.993
Actinotignum schaalii	-0.018	0.289	0.950	0.993
Negativicoccus	-0.010	0.229	0.965	0.994
Corynebacterium aurimucosum/minutissimum/singulare	0.007	0.267	0.978	0.994
Campylobacter hominis	-0.005	0.256	0.983	0.994
Corynebacterium tuberculostearicum	0.002	0.311	0.994	0.994

© 2022 Srinivasan S et al. JAMA Network Open.

Supplement 2, eTable 2: Comparison of change in individual bacterial taxa in the moisturizer group versus the placebo group

Taxon	Moisturizer (Est)	Moisturizer (SE)	Moisturizer (p-value)	Moisturizer (q-value)
Streptococcus mitis group	-1.788	0.467	<0.001	0.019
Anaerococcus vaginalis	-1.133	0.410	0.006	0.149
Enterococcus faecalis	0.949	0.327	0.004	0.149
Corynebacterium simulans/striatum	0.776	0.273	0.005	0.149
Corynebacterium amycolatum	0.866	0.324	0.008	0.156
Staphylococcus capitis/caprae/epidermidis	0.790	0.303	0.010	0.159
Lactobacillus acidophilus/kitasatonis	0.708	0.287	0.015	0.172
Prevotella timonensis	-1.100	0.441	0.014	0.172
Streptococcus agalactiae	1.364	0.564	0.017	0.174
Peptoniphilus coxii	-0.669	0.285	0.020	0.186
Peptoniphilus koenoeneniae	0.550	0.238	0.022	0.186
Dialister propionicifaciens	-0.771	0.356	0.032	0.249
Corynebacterium riegelii	0.478	0.244	0.052	0.375
Negativicoccus	0.414	0.225	0.068	0.453
Ezakiella	-0.604	0.335	0.074	0.456
Actinomyces europaeus	0.401	0.240	0.097	0.561
Peptoniphilus lacrimalis	-0.390	0.244	0.113	0.618
Varibaculum anthropi/cambriense	-0.591	0.378	0.120	0.622
Peptoniphilus grossensis/harei/phoceensis	-0.565	0.395	0.155	0.635
Campylobacter ureolyticus	-0.428	0.281	0.130	0.635
Corynebacterium pyruviciproducens	-0.393	0.276	0.157	0.635
Mobiluncus curtisii	-0.380	0.266	0.154	0.635
Prevotella disiens	-0.581	0.394	0.142	0.635
Atopobium vaginae	0.658	0.492	0.183	0.653
Peptoniphilus	-0.456	0.332	0.172	0.653
Lactobacillus crispatus	0.847	0.628	0.180	0.653
Dialister micraerophilus	-0.468	0.359	0.195	0.670
Lactobacillus casei group	0.342	0.282	0.227	0.705
Casaltella massiliensis	-0.299	0.247	0.227	0.705
Porphyromonas somerae	0.305	0.249	0.223	0.705
Lactobacillus gasseri/johnsonii	0.621	0.558	0.268	0.716
Anaerococcus mediterraneensis	-0.378	0.377	0.318	0.716
Lactobacillus jensenii	0.561	0.510	0.274	0.716
Prevotella bivia	0.366	0.343	0.288	0.716
Facklamia ignava	0.231	0.237	0.331	0.716
Prevotella bergensis	-0.242	0.245	0.326	0.716
Gemella haemolysans/sanguinis	-0.317	0.282	0.264	0.716
Lactobacillus reuteri/vaginalis	0.405	0.377	0.285	0.716
Actinomyces turicensis	-0.320	0.321	0.321	0.716
Escherichia coli	0.350	0.349	0.318	0.716
Candidatus Peptoniphilus massiliensis	0.247	0.227	0.278	0.716
Staphylococcus haemolyticus	0.271	0.261	0.300	0.716
Corynebacterium	0.383	0.329	0.246	0.716
Porphyromonas uenonis	-0.254	0.284	0.373	0.738
Faecalibacterium prausnitzii	-0.236	0.262	0.369	0.738
Porphyromonas asaccharolytica	-0.214	0.238	0.369	0.738
Fastidiosipila sanguinis	0.210	0.224	0.350	0.738
Lagierella	0.212	0.242	0.384	0.744
Prevotella buccalis	-0.187	0.238	0.433	0.747
Fusobacterium nucleatum	-0.203	0.258	0.434	0.747
Fenollaria massiliensis/timonensis	-0.227	0.282	0.423	0.747
Corynebacterium sundsvallense	0.229	0.285	0.422	0.747
Anaerococcus	0.254	0.304	0.404	0.747
Corynebacterium aurimucosum/minutissimum/singulare	0.209	0.262	0.428	0.747
Bifidobacterium bifidum	0.203	0.302	0.503	0.796
Bifidobacterium breve	0.210	0.324	0.518	0.796
Alloscardovia omnicolens	0.266	0.403	0.509	0.796
Howardella	-0.252	0.379	0.508	0.796
Peptostreptococcus anaerobius	-0.179	0.279	0.522	0.796
Atopobium deltae	-0.163	0.251	0.516	0.796
Anaerococcus prevotii/tetradius	-0.194	0.299	0.518	0.796
Prevotella colorans	0.139	0.223	0.534	0.798
Aerococcus christensenii	0.197	0.327	0.549	0.798
Varibaculum	-0.128	0.213	0.548	0.798
Bifidobacterium dentium	0.204	0.367	0.579	0.807
Facklamia hominis	-0.165	0.299	0.582	0.807
Porphyromonas bennonis	-0.135	0.235	0.567	0.807
Corynebacterium genitalium	0.115	0.229	0.615	0.818
Anaerococcus senegalensis	-0.138	0.261	0.598	0.818
Corynebacterium tuberculostearicum	0.156	0.306	0.610	0.818
Fenollaria	0.110	0.267	0.682	0.882
Arcanobacterium	0.096	0.234	0.683	0.882
Anaerococcus lactolyticus	-0.093	0.267	0.728	0.928

© 2022 Srinivasan S et al. JAMA Network Open.

Taxon	Moisturizer (Est)	Moisturizer (SE)	Moisturizer (p-value)	Moisturizer (q-value)
Actinomyces neuii	-0.137	0.417	0.742	0.933
Finegoldia magna	-0.099	0.398	0.803	0.941
Lawsonella	0.085	0.305	0.780	0.941
Streptococcus anginosus group	-0.139	0.610	0.820	0.941
Lactobacillus iners	-0.178	0.732	0.808	0.941
Dialister sp. type 2	-0.112	0.385	0.772	0.941
Anaerococcus hydrogenalis/rubeinfantis	0.071	0.304	0.815	0.941
Actinobaculum massiliense	-0.081	0.294	0.783	0.941
Bifidobacterium longum	0.049	0.272	0.859	0.951
Prevotella corporis	0.058	0.308	0.852	0.951
Actinotignum schaalii	-0.054	0.284	0.851	0.951
Corynebacterium pseudogenitalium/tuberculostearicum	-0.048	0.324	0.882	0.965
Corynebacterium mycetoides	-0.027	0.271	0.920	0.969
Gardnerella vaginalis	-0.052	0.668	0.938	0.969
Parvimonas	-0.034	0.258	0.897	0.969
Murdochiella asaccharolytica/Levyella massiliensis	-0.022	0.234	0.926	0.969
Campylobacter hominis	-0.021	0.251	0.933	0.969
Corynebacterium coyleae	0.017	0.258	0.948	0.969
Helcococcus	-0.006	0.251	0.981	0.985
Ureaplasma parvum/urealyticum	0.006	0.338	0.985	0.985

© 2022 Srinivasan S et al. JAMA Network Open.

Supplement 2, eTable 3: Comparison of change in individual metabolites in the estradiol group versus the placebo group

Metabolite	Estradiol (Estimate)	Estradiol (SE)	Estradiol (p-value)	Estradiol (q-value)
Methionine	1.185	0.211	<0.001	<0.001
ethanolamine	0.903	0.172	<0.001	<0.001
Cytosine	2.065	0.404	<0.001	<0.001
Guanosine	1.369	0.280	<0.001	<0.001
N-Carbamoyl-B-Alanine	0.933	0.200	<0.001	<0.001
NADH	0.914	0.224	<0.001	0.001
Indole-3-Lactate	1.114	0.274	<0.001	0.001
5'-methylthioadenosine	0.888	0.219	<0.001	0.001
Leucine /D-norleucine	0.801	0.206	<0.001	0.001
Tryptophan	0.752	0.200	<0.001	0.002
Uridine	1.028	0.278	<0.001	0.002
iso-Leucine /allo-isoleucine	0.772	0.210	<0.001	0.002
Hypoxanthine	0.829	0.226	<0.001	0.002
Hydroxyproline	0.311	0.088	0.001	0.003
2-Aminoadipate	0.668	0.190	0.001	0.003
phenyllactic acid	1.533	0.445	0.001	0.003
pseudouridine	0.512	0.149	0.001	0.003
Mevalonate	1.068	0.315	0.001	0.004
N-Ac-Glutamate	0.470	0.140	0.001	0.004
lactate	0.588	0.182	0.002	0.005
Sarcosine	0.215	0.067	0.002	0.005
Adenine	1.615	0.503	0.002	0.005
riboflavin	0.736	0.229	0.002	0.005
Tyramine	1.871	0.608	0.003	0.008
Alanine	0.197	0.064	0.003	0.008
Phenylalanine	0.562	0.189	0.003	0.010
Mannose	0.579	0.201	0.005	0.013
Valine	0.752	0.264	0.005	0.014
Pentothenate	0.611	0.215	0.005	0.014
N-Ac-Alanine	0.448	0.179	0.014	0.035
arabitol/xylitol	0.418	0.169	0.014	0.036
Asparagine	0.422	0.172	0.015	0.038
Glycine	0.295	0.127	0.022	0.051
succinylcarnitine	0.422	0.188	0.026	0.058
Cystine	0.419	0.189	0.028	0.061
N-Acetylneuraminate	0.466	0.211	0.029	0.063
Threonine	0.273	0.127	0.033	0.068
PPA	0.429	0.201	0.035	0.070
N-Ac-Tyrosine	0.306	0.145	0.037	0.074
Uracil	0.744	0.370	0.046	0.091
homoarginine	0.509	0.255	0.047	0.091
2-Hydroxyisovaleric Acid	-0.167	0.069	0.016	0.040
L-Kynurenine	-0.213	0.104	0.042	0.083
N-Ac-Tryptophan	-0.324	0.112	0.004	0.012
cis-aconitate	-0.383	0.102	0.000	0.002
phenylacetic acid	-0.420	0.191	0.030	0.064
Acetylcarnitine	-0.420	0.172	0.016	0.038
Acetoacetate	-0.442	0.204	0.032	0.066
PGE	-0.449	0.118	0.000	0.001
2-hydroxybutyrate	-0.452	0.228	0.049	0.094
gentisate	-0.452	0.135	0.001	0.004
2-hydroxyphenylacetate	-0.469	0.208	0.025	0.057
Glycochenodeoxycholate	-0.477	0.128	0.000	0.002
Glycerol-3-P	-0.478	0.211	0.025	0.057
indole-3-carboxylic acid	-0.493	0.205	0.017	0.041
o-phosphoethanolamine	-0.494	0.162	0.003	0.008
threonic/erythronic acid	-0.497	0.119	0.000	0.001
3/4-hydroxyphenylacetic acid	-0.504	0.223	0.025	0.057
biliverdin	-0.512	0.139	0.000	0.002
N-Acetyl-Aspartate (naa)	-0.523	0.158	0.001	0.004
Kynurenic Acid	-0.546	0.147	0.000	0.002
Sucrose	-0.550	0.275	0.047	0.091
Indole-3-Acetic Acid	-0.556	0.197	0.005	0.014
Urate	-0.562	0.136	0.000	0.001
Citrulline	-0.566	0.135	0.000	0.001
Adipic Acid	-0.572	0.160	0.000	0.002
hydrocinnamic acid	-0.575	0.179	0.002	0.005
Linoleic Acid	-0.610	0.186	0.001	0.005

© 2022 Srinivasan S et al. JAMA Network Open.

Metabolite	Estradiol (Estimate)	Estradiol (SE)	Estradiol (p-value)	Estradiol (q-value)
Citraconic Acid	-0.618	0.185	0.001	0.004
Alpha-Ketoglutaric Acid	-0.625	0.154	0.000	0.001
Orotate	-0.634	0.204	0.002	0.007
3-hydroxyisovaleric acid	-0.635	0.174	0.000	0.002
xylose	-0.641	0.183	0.001	0.003
Fumaric Acid	-0.641	0.177	0.000	0.002
myo-inositol	-0.660	0.141	0.000	0.000
5-Aminovaleric Acid	-0.696	0.169	0.000	0.001
2-oxo-isocaproic acid	-0.705	0.237	0.003	0.010
Hippuric Acid	-0.715	0.329	0.032	0.066
Xanthine	-0.734	0.284	0.011	0.028
Inositol	-0.846	0.219	0.000	0.001
Arachidonate	-0.858	0.200	0.000	0.000
Azelaic Acid	-0.878	0.198	0.000	0.000
Glutaric Acid	-0.922	0.206	0.000	0.000
4-hydroxybenzoic acid	-0.964	0.364	0.009	0.024
Allantoin	-1.008	0.250	0.000	0.001
Margaric Acid	-1.103	0.185	0.000	0.000
AMP	-1.119	0.250	0.000	0.000
Xanthosine	-1.309	0.317	0.000	0.001
IMP	-1.442	0.313	0.000	0.000
Niacinamide	-1.521	0.398	0.000	0.001
isovalerylcarnitine	-0.374	0.190	0.051	0.095
DCMP	0.433	0.221	0.051	0.095
3-methyl-3-hydroxyglutaric acid	-0.253	0.130	0.054	0.099
Histamine	0.762	0.401	0.060	0.107
N6-Acetyl-lysine	-0.342	0.180	0.060	0.107
Glutamic acid	0.214	0.114	0.062	0.110
Reduced glutathione	0.606	0.327	0.066	0.117
Proline	0.324	0.177	0.069	0.120
alpha-ketophenylacetic acid	-0.423	0.232	0.071	0.122
Thymine	0.511	0.285	0.075	0.128
Nicotinic Acid	0.552	0.310	0.077	0.130
Carnitine	-0.175	0.098	0.078	0.131
Sorbitol	0.320	0.182	0.081	0.135
mannitol	0.357	0.205	0.083	0.137
NAD	0.377	0.221	0.090	0.147
SAH	0.345	0.208	0.099	0.160
Cadaverine	0.409	0.249	0.103	0.165
deoxycholic acid	-0.059	0.037	0.113	0.178
N-AcetylGlycine	0.144	0.092	0.118	0.186
CMP	-0.263	0.171	0.127	0.196
n-isovalerylglycine	-0.254	0.165	0.127	0.196
Arginine	-0.318	0.208	0.130	0.198
n-formylmethionine	-0.177	0.117	0.132	0.200
D-Leucic Acid	0.450	0.300	0.135	0.202
4-Pyridoxic acid	-0.450	0.300	0.136	0.202
3-Indoxyl Sulfate	-0.384	0.266	0.150	0.220
Tyrosine	-0.173	0.120	0.151	0.220
dimethylarginine	-0.202	0.142	0.158	0.229
3HBA	-0.334	0.243	0.172	0.247
2-oxoisovalerrate	-0.364	0.271	0.180	0.255
Cystathionine	-0.096	0.071	0.181	0.255
Malondialdehyde	-0.365	0.274	0.185	0.259
Aspartic Acid	0.181	0.137	0.189	0.263
Choline	-0.183	0.140	0.194	0.267
Serine	-0.134	0.103	0.195	0.267
Adenosine	0.246	0.193	0.206	0.280
5-methyluridine	0.192	0.155	0.219	0.295
indole-3-propionate	-0.336	0.277	0.228	0.304
7-methylguanine	0.235	0.197	0.235	0.312
L-mandelic acid	-0.295	0.256	0.251	0.330
trigonelline	-0.361	0.316	0.256	0.334
Linolenic Acid	0.122	0.109	0.264	0.341
Putrescine	0.324	0.297	0.276	0.355
gama-Aminobutyrate	0.184	0.170	0.280	0.358
UDP-GlcNAc	0.136	0.131	0.300	0.378
beta alanine	-0.239	0.230	0.301	0.378
1-Methyladenosine	0.210	0.204	0.303	0.378

© 2022 Srinivasan S et al. JAMA Network Open.

Metabolite	Estradiol (Estimate)	Estradiol (SE)	Estradiol (p-value)	Estradiol (q-value)
Oxidized glutathione	-0.219	0.220	0.321	0.398
Creatine	0.080	0.086	0.349	0.430
N-Ac-L-Glutamine	0.134	0.143	0.352	0.430
Pyruvate	0.277	0.301	0.359	0.436
6-Methyladenosine	-0.089	0.103	0.390	0.470
Oxalacetate	0.118	0.139	0.397	0.475
Lysine	-0.138	0.172	0.425	0.505
Glyceraldehyde	-0.110	0.142	0.440	0.519
Glucoronate	0.175	0.230	0.448	0.525
2-Hydroxyglutarate	0.113	0.150	0.455	0.529
Methyl-OH-isobutyrate	0.200	0.271	0.461	0.533
Succinate	0.190	0.264	0.473	0.542
phenylacetylglutamine	-0.194	0.304	0.524	0.597
Glucose	-0.147	0.239	0.539	0.610
Indole	0.019	0.035	0.584	0.657
Inosine	0.189	0.356	0.597	0.668
glutarylcarnitine	-0.083	0.173	0.632	0.702
N6-Trimethyllysine	-0.067	0.144	0.641	0.707
Taurine	0.050	0.133	0.711	0.779
Cotinine	0.075	0.208	0.718	0.782
Histidine	-0.041	0.121	0.731	0.792
3-Hydroxykynurenine	-0.138	0.426	0.747	0.803
Cytidine	-0.029	0.096	0.768	0.817
S-methylcysteine	-0.039	0.133	0.769	0.817
Glutamine	0.027	0.109	0.803	0.847
Ornithine	0.048	0.216	0.824	0.864
methionine sulfoxide	-0.061	0.287	0.832	0.868
Trimethylamine-N-oxide	-0.052	0.281	0.854	0.885
Deoxycarnitine	0.037	0.212	0.862	0.888
1/3-Methylhistidine	0.031	0.211	0.882	0.903
Glycerate	0.015	0.131	0.910	0.926
N2,N2-Dimethylguanosine	0.015	0.229	0.948	0.959
Creatinine	-0.007	0.184	0.971	0.976
Cysteinyl-Glycine (Cys-Gly)	0.001	0.103	0.989	0.989

© 2022 Srinivasan S et al. JAMA Network Open.

Supplement 2, eTable 4: Comparison of change in individual metabolites in the moisturizer group versus the placebo group

Metabolite	Moisturizer (Estimate)	Moisturizer (SE)	Moisturizer (p-value)	Moisturizer (q-value)
5-Aminovaleric Acid	-0.464	0.166	0.006	0.979
N6-Acetyl-lysine	-0.397	0.177	0.027	0.979
2-Aminoadipate	0.380	0.187	0.044	0.979
PGE	-0.233	0.116	0.047	0.979
Arginine	-0.410	0.205	0.048	0.979
L-Kynurenine	0.194	0.102	0.060	0.979
7-methylguanine	-0.309	0.194	0.112	0.979
1/3-Methylhistidine	-0.329	0.207	0.116	0.979
N-Ac-Alanine	-0.278	0.176	0.117	0.979
UDP-GlcNAc	0.194	0.129	0.135	0.979
Creatine	0.126	0.084	0.137	0.979
homoarginine	-0.372	0.250	0.139	0.979
NAD	-0.315	0.217	0.149	0.979
Guanosine	0.399	0.276	0.151	0.979
Glycerol-3-P	0.299	0.208	0.153	0.979
3-Hydroxykynurenine	-0.600	0.418	0.154	0.979
Adenosine	0.271	0.190	0.157	0.979
Citrulline	-0.179	0.132	0.178	0.979
Carnitine	0.129	0.097	0.183	0.979
Methyl-OH-isobutyrate	0.356	0.266	0.183	0.979
Hippuric Acid	0.427	0.324	0.190	0.979
Hypoxanthine	0.291	0.222	0.192	0.979
Glycerate	-0.168	0.129	0.194	0.979
indole-3-propionate	0.352	0.273	0.199	0.979
2-hydroxybutyrate	-0.286	0.224	0.204	0.979
Uridine	0.345	0.273	0.208	0.979
Succinate	0.327	0.259	0.210	0.979
3/4-hydroxyphenylacetic acid	-0.267	0.219	0.225	0.979
Mevalonate	0.375	0.310	0.228	0.979
Margaric Acid	-0.219	0.182	0.230	0.979
N6-Trimethyllysine	-0.169	0.141	0.234	0.979
indole-3-carboxylic acid	0.239	0.201	0.238	0.979
N-AcetylGlycine	0.107	0.090	0.239	0.979
Xanthosine	-0.367	0.311	0.240	0.979
6-Methyladenosine	0.117	0.102	0.250	0.979
Nicotinic Acid	-0.350	0.304	0.253	0.979
Valine	-0.296	0.260	0.257	0.979
AMP	0.278	0.246	0.260	0.979
Inositol	-0.231	0.215	0.286	0.979
N2,N2-Dimethylguanosine	-0.239	0.225	0.291	0.979
Inosine	0.353	0.351	0.315	0.979
2-oxoisovalerrate	0.267	0.266	0.318	0.979
Glycochenodeoxycholate	0.126	0.126	0.320	0.979
phenylacetylglutamine	0.297	0.299	0.322	0.979
gentisate	0.129	0.133	0.332	0.979
Tryptophan	-0.188	0.197	0.340	0.979
Choline	-0.124	0.137	0.368	0.979
3-Indoxyl Sulfate	0.233	0.261	0.374	0.979
2-Hydroxyisovaleric Acid	0.059	0.067	0.379	0.979
Sucrose	0.238	0.271	0.380	0.979
2-hydroxyphenylacetate	0.177	0.204	0.387	0.979
Linolenic Acid	0.093	0.107	0.389	0.979
Arachidonate	0.168	0.197	0.393	0.979
Allantoin	-0.209	0.245	0.395	0.979
D-Leucic Acid	-0.248	0.295	0.402	0.979
Sorbitol	0.146	0.179	0.416	0.979
gamma-Aminobutyrate	0.134	0.167	0.423	0.979
phenyllactic acid	-0.342	0.438	0.436	0.979
methionine sulfoxide	-0.216	0.282	0.446	0.979
Aspartic Acid	0.102	0.135	0.449	0.979
Reduced glutathione	0.243	0.322	0.452	0.979
Niacinamide	0.295	0.391	0.452	0.979
Pyruvate	-0.218	0.296	0.463	0.979
L-mandelic acid	-0.180	0.251	0.475	0.979
Cadaverine	-0.175	0.245	0.475	0.979
n-formylmethionine	0.079	0.115	0.491	0.979
Hydroxyproline	0.060	0.087	0.494	0.979
Alpha-Ketoglutaric Acid	-0.103	0.152	0.498	0.979
myo-inositol	-0.093	0.138	0.503	0.979
3-methyl-3-hydroxyglutaric acid	0.085	0.128	0.505	0.979
N-Ac-Tryptophan	0.073	0.110	0.507	0.979
Orotate	0.132	0.200	0.510	0.979
2-Hydroxyglutarate	-0.097	0.148	0.513	0.979

Metabolite	Moisturizer (Estimate)	Moisturizer (SE)	Moisturizer (p-value)	Moisturizer (q-value)
Cystathionine	-0.046	0.070	0.514	0.979
Cystine	0.121	0.185	0.515	0.979
alpha-ketophenylacetic acid	-0.146	0.229	0.523	0.979
deoxycholic acid	0.023	0.036	0.524	0.979
Ornithine	0.135	0.212	0.527	0.979
dimethylarginine	-0.086	0.140	0.542	0.979
Indole-3-Lactate	-0.164	0.270	0.545	0.979
Azelaic Acid	-0.117	0.195	0.549	0.979
trigonelline	-0.181	0.311	0.561	0.979
Malondialdehyde	0.156	0.269	0.564	0.979
biliverdin	0.078	0.136	0.569	0.979
N-Carbamoyl-B-Alanine	-0.111	0.197	0.573	0.979
glutarylcarnitine	-0.094	0.170	0.580	0.979
Indole-3-Acetic Acid	0.107	0.193	0.580	0.979
Acetylcarnitine	0.093	0.169	0.581	0.979
Proline	-0.092	0.174	0.597	0.979
Putrescine	0.150	0.292	0.608	0.979
IMP	0.158	0.308	0.610	0.979
Serine	0.051	0.101	0.615	0.979
Uracil	0.182	0.364	0.618	0.979
Glucose	-0.117	0.235	0.620	0.979
3HBA	-0.117	0.239	0.626	0.979
Glycine	0.061	0.125	0.627	0.979
N-Acetyl-Aspartate (naa)	0.075	0.155	0.628	0.979
Xanthine	-0.135	0.280	0.630	0.979
o-phosphoethanolamine	0.076	0.160	0.635	0.979
iso-Leucine /allo-isoleucine	-0.097	0.207	0.640	0.979
n-isovalerylglycine	0.075	0.163	0.643	0.979
pseudouridine	-0.068	0.147	0.646	0.979
Thymine	-0.128	0.280	0.648	0.979
Phenylalanine	-0.085	0.186	0.649	0.979
N-Acetylneuraminate	-0.094	0.208	0.652	0.979
Fumaric Acid	0.079	0.174	0.653	0.979
Citraconic Acid	0.082	0.181	0.653	0.979
ethanolamine	-0.072	0.169	0.670	0.979
Glutamine	0.045	0.107	0.673	0.979
Glyceraldehyde	0.058	0.140	0.677	0.979
N-Ac-Glutamate	-0.056	0.137	0.682	0.979
Adipic Acid	0.064	0.157	0.686	0.979
4-Pyridoxic acid	0.119	0.295	0.686	0.979
Histamine	-0.153	0.395	0.699	0.979
N-Ac-L-Glutamine	0.054	0.141	0.703	0.979
isovalerylcarnitine	0.071	0.186	0.705	0.979
Deoxycarnitine	-0.078	0.209	0.709	0.979
Glucoronate	0.084	0.226	0.711	0.979
SAH	-0.075	0.204	0.714	0.979
Oxidized glutathione	0.077	0.217	0.721	0.979
Leucine /D-norleucine	-0.072	0.203	0.723	0.979
xylose	-0.064	0.180	0.724	0.979
Tyrosine	-0.040	0.118	0.733	0.979
1-Methyladenosine	0.066	0.200	0.743	0.979
5-methyluridine	-0.050	0.153	0.745	0.979
3-hydroxyisovaleric acid	0.055	0.171	0.748	0.979
hydrocinnamic acid	0.057	0.176	0.748	0.979
Acetoacetate	0.064	0.201	0.752	0.979
Cytidine	-0.028	0.095	0.770	0.979
Cytosine	0.115	0.397	0.773	0.979
Threonine	0.036	0.125	0.776	0.979
Trimethylamine-N-oxide (TMAO)	-0.078	0.276	0.779	0.979
5'-methylthioadenosine	0.059	0.215	0.784	0.979
Lysine	-0.045	0.169	0.792	0.979
mannitol	0.053	0.201	0.793	0.979
DCMP	-0.056	0.217	0.797	0.979
CMP	-0.042	0.169	0.802	0.979
N-Ac-Tyrosine	0.034	0.143	0.812	0.979
beta alanine	-0.053	0.226	0.816	0.979
Linoleic Acid	-0.042	0.183	0.819	0.979
Creatinine	-0.036	0.181	0.842	0.979
Pentothenate	0.036	0.212	0.864	0.979
PPA	-0.034	0.198	0.864	0.979
Asparagine	0.029	0.169	0.864	0.979
Mannose	0.034	0.198	0.865	0.979
S-methylcysteine	-0.022	0.131	0.867	0.979
arabitol/xylitol	-0.028	0.166	0.867	0.979

© 2022 Srinivasan S et al. JAMA Network Open.

Metabolite	Moisturizer (Estimate)	Moisturizer (SE)	Moisturizer (p-value)	Moisturizer (q-value)
Methionine	-0.035	0.208	0.868	0.979
Cysteinyl-Glycine (Cys-Gly)	0.017	0.101	0.869	0.979
cis-aconitate	-0.016	0.100	0.870	0.979
4-hydroxybenzoic acid	0.057	0.358	0.874	0.979
Cotinine	-0.032	0.204	0.876	0.979
NADH	-0.032	0.220	0.883	0.979
Alanine	0.009	0.063	0.885	0.979
phenylacetic acid	0.027	0.188	0.888	0.979
Kynurenic Acid	0.018	0.145	0.901	0.985
Adenine	0.054	0.494	0.913	0.985
Indole	0.003	0.034	0.922	0.985
Oxalacetate	-0.011	0.137	0.934	0.985
Glutaric Acid	0.014	0.202	0.945	0.985
threonic/erythronic acid	-0.008	0.117	0.946	0.985
Histidine	0.008	0.119	0.949	0.985
Urate	-0.008	0.134	0.952	0.985
riboflavin	-0.013	0.225	0.954	0.985
Sarcosine	0.003	0.066	0.960	0.985
2-oxo-isocaproic acid	-0.011	0.233	0.961	0.985
Glutamic acid	0.004	0.112	0.970	0.985
Tyramine	-0.022	0.598	0.970	0.985
Taurine	0.004	0.131	0.976	0.985
lactate	-0.005	0.179	0.979	0.985
succinylcarnitine	0.001	0.185	0.995	0.995

© 2022 Srinivasan S et al. JAMA Network Open.

Supplement 2 eTable 5: Demographic and clinical characterisitics of participants by microbial diversity grouping at baseline

	Low diversity (n = 59)	High Diversity (n = 68)	*P* -value
Age	60 ± 4	61 ± 4	0.23
Arm			0.96
Estradiol	17 (29)	21 (30)	
Moisturizer	22 (37)	24 (35)	
Placebo	20 (34)	24 (35)	
Race			0.03
White	49 (83)	64 (93)	
Black	6 (10)	0 (0)	
Other	4 (7)	5 (7)	
MBS			0.38
Dryness	12 (20)	18 (26)	
Pain with sex	32 (54)	39 (57)	
Itch/Burn/Irritation	15 (26)	11 (17)	
VMI < 5% superficial	49/57 (86)	54/55 (98)	**0.02**
[a]pH	6.2 ± 1.1	7.2 ± 0.6	**< 0.001**
[ab]Serum estradiol (pg/mL)	3.41 ± 1.76	3.78 ± 1.95	0.227
[a]SDI	0.66 ± 0.56	2.27 ± 0.96	**< 0.001**
Lactobacillus/Bifidobacterium dominance	35 (59)	3 (4)	**< 0.001**
Subsequent symptom improvement ≥ 2 points	29 (49)	35 (51)	0.79

[a]Mean ± standard deviation

[b]Includes only women in the placebo and estradiol groups for whom we measured serum estradiol

© 2022 Srinivasan S et al. JAMA Network Open.
