# Supplementary material for: Impact of Topical Interventions on the Vaginal Microbiota and Metabolome in Postmenopausal Women: A Secondary Analysis of a Randomized Clinical Trial
Source: JAMA Netw Open. 2022 Mar 30;5(3):e225032. doi: 10.1001/jamanetworkopen.2022.5032 (PMC8968546; doi:10.1001/jamanetworkopen.2022.5032)
Supplement: Supplement 3. — Data Sharing Statement [file jamanetwopen-e225032-s003.pdf]

Data Sharing Statement

Srinivasan. Impact of Topical Interventions on the Vaginal Microbiota and Metabolome in Postmenopausal Women. *JAMA Netw Open*. Published March 30, 2022. doi:10.1001/jamanetworkopen.2022.5032

Data
Data available: Yes
Data types: Deidentified participant data, Data dictionary
How to access data: The MsFLASH Data Coordinating Center (DCC) has a National Institute of Aging (NIA)-approved Data Sharing Plan. The DCC has datasets prepared with all data elements with certain deletions and recoding to protect study participant confidentiality. These datasets include documentation in electronic form. Dr. Guthrie, the study PI, is the primary contact.
When available: With publication

Supporting Documents
Document types: None

Additional Information
Data Sharing Policy for the MsFLASH Network (revised February 2, 2018)
MsFLASH Network policies, procedures, and trial protocols will be developed to support data sharing both within and outside of the network in accordance with NIH policies as outlined at https://grants.nih.gov/grants/policy/data_sharing. The study consent form, HIPAA authorization and IRB documentation will be written to permit sharing of de-identified data and to indicate that the participant's data will be shared with other qualified researchers as mandated by policy from the National Institutes of Health. All data required by protocol will be centrally managed in the MsFLASH database while there are funds to support the DCC. Upon completion of a trial and after all appropriate quality checks have been applied, these data will be extracted from the central database and an analytic SAS dataset created which will serve as the basis for all publications from the trial. The DCC will perform the statistical analyses in collaboration with the Protocol Chair for the publication of the primary results. The DCC will also provide analytic support to MsFLASH investigators for secondary outcome papers. Once the manuscript(s) of primary results has been accepted for publication, the SAS dataset will be de-identified and made available to all MsFLASH investigators for their use within the policies and procedures developed by the Network.
After each trial's main results are accepted for publication, the DCC will prepare limited access datasets that include all data elements in the main publication with certain deletions and recoding to protect study subject confidentiality. Data from any ancillary studies funded by the NIH will also be made available. The DCC will remove any participant, site or other sensitive information from the final database, including removal of all Personal Health Information (PHI) and indirect identifiers that are not listed as PHI but could lead to "deductive disclosure," such as comment fields and site numbers. The limited access datasets will include substantial documentation in electronic form, including data collection forms, study protocols and procedures, data dictionaries, and a list of major publications using these data. These inclusions are all designed to facilitate use of the data by persons who are not intimately familiar with the study. We envision making documented datasets available to the scientific community soon after publication of the primary trial reports. Once MsFLASH funding has expired, datasets will be supplied to the NIA and applications will be reviewed by the NIA Project Scientist.
An outside investigator requesting study data will complete a manuscript proposal, providing the specific research question being considered, an overview of the methodology to be applied, and academic titles/mentors of the investigators. The MsFLASH PIs will review these

proposals and accept those from qualified investigators. Clearly frivolous requests from unqualified investigators will be screened out. The proposal will then be sent to Network investigators for enlistment of a study sponsor, that is, an investigator with knowledge of the MsFLASH studies that can serve as a co-author. As outlined in the NIH Data Sharing Policy, investigators must sign a Data Use Agreement before obtaining access to datasets. Any reports, abstracts, presentations and manuscripts should acknowledge that funding was provided by the National Institute on Aging.
